# Translational regulation of SND1 governs endothelial homeostasis during stress

**DOI:** 10.1172/JCI168730

**Published:** 2025-02-03

**Authors:** Zhenbo Han, Gege Yan, Jordan Jousma, Sarath Babu Nukala, Mehdi Amiri, Stephen Kiniry, Negar Tabatabaei, Youjeong Kwon, Sen Zhang, Jalees Rehman, Sandra Pinho, Sang-Bing Ong, Pavel V. Baranov, Soroush Tahmasebi, Sang-Ging Ong

**Affiliations:** 1 Department of Pharmacology & Regenerative Medicine, University of Illinois College of Medicine, Chicago, Illinois, USA.; 2 Department of Biochemistry, McGill University, Montreal, Quebec, Canada.; 3 School of Biochemistry and Cell Biology, University College Cork, Cork, Ireland.; 4 University of Illinois Cancer Center, Chicago, Illinois, USA.; 5 Department of Medicine and Therapeutics, Faculty of Medicine, Chinese University of Hong Kong (CUHK), Hong Kong SAR, China.; 6 Centre for Cardiovascular Genomics and Medicine (CCGM), Lui Che Woo Institute of Innovative Medicine, CUHK, Hong Kong SAR, China.; 7 Hong Kong Hub of Pediatric Excellence (HK HOPE), Hong Kong Children’s Hospital (HKCH), Hong Kong SAR, China.; 8 Kunming Institute of Zoology — The Chinese University of Hong Kong (KIZ-CUHK) Joint Laboratory of Bioresources and Molecular Research of Common Diseases, Kunming Institute of Zoology, Chinese Academy of Sciences, Kunming, China.; 9 Division of Cardiology, Department of Medicine, University of Illinois College of Medicine, Chicago, Illinois, USA.

**Keywords:** Vascular biology, Cancer, Cardiovascular disease, Endothelial cells

## Abstract

Translational control shapes the proteome and is particularly important in regulating gene expression under stress. A key source of endothelial stress is treatment with tyrosine kinase inhibitors (TKIs), which lowers cancer mortality but increases cardiovascular mortality. Using a human induced pluripotent stem cell–derived endothelial cell (hiPSC-EC) model of sunitinib-induced vascular dysfunction combined with ribosome profiling, we assessed the role of translational control in hiPSC-ECs in response to stress. We identified staphylococcal nuclease and tudor domain–containing protein 1 (SND1) as a sunitinib-dependent translationally repressed gene. SND1 translational repression was mediated by the mTORC1/4E-BP1 pathway. SND1 inhibition led to endothelial dysfunction, whereas SND1 OE protected against sunitinib-induced endothelial dysfunction. Mechanistically, SND1 transcriptionally regulated UBE2N, an E2-conjugating enzyme that mediates K63-linked ubiquitination. UBE2N along with the E3 ligases RNF8 and RNF168 regulated the DNA damage repair response pathway to mitigate the deleterious effects of sunitinib. In silico analysis of FDA-approved drugs led to the identification of an ACE inhibitor, ramipril, that protected against sunitinib-induced vascular dysfunction in vitro and in vivo, all while preserving the efficacy of cancer therapy. Our study established a central role for translational control of SND1 in sunitinib-induced endothelial dysfunction that could potentially be therapeutically targeted to reduce sunitinib-induced vascular toxicity.

## Abstract 2

<span>Translational control of SND1 mediates sunitinib-induced vascular toxicity and may serves as a therapeutic target.</span>

## Introduction

Translational control is a key regulatory mechanism that controls protein abundance and thereby dictates identity and functions across cell types and tissues (1, 2). Although many studies have focused on transcriptional regulation, much less is known regarding mRNA translational regulation (3). The transcriptional level alone cannot directly determine protein abundance, especially during the dynamic transitional state, in which a temporal delay exists between transcription and translation (4, 5). Thus, analysis of translation complements the study of transcription in predicting the final expression of genes. Ribosome profiling (Ribo-Seq) is a high-throughput method that enables a genome-wide quantitative analysis of translation based on deep sequencing of ribosome-protected mRNA fragments (6, 7). With this strategy, the rate of protein synthesis can be determined by quantifying the density of protected fragments on the corresponding transcript.

The vasculature is lined by endothelial cells (ECs) (8) with remarkable endothelial heterogeneity across distinct tissues and organs (9, 10), in part dictated by signals from the tissue microenvironment (11). Additionally, a range of transcription factors (TFs) regulate EC homeostasis, leading to cell-specific alterations in gene expression (12). Although the field of transcriptome analysis has greatly expanded our understanding of endothelial biology, the extent to which mRNA translational control contributes to the dynamic regulation of ECs, especially under conditions of stress, remains less well characterized (13, 14).

In the present study, we investigated the molecular mechanisms by which sunitinib, a tyrosine kinase inhibitor (TKI), induces endothelial dysfunction with a focus on translational regulation. Over the past few decades, there has been a significant decline in cancer-related mortality, partly attributed to the development of molecularly targeted therapies (15, 16). Unfortunately, the success of these drugs, including TKIs, has been tempered by a concomitant rise in the prevalence of cardiotoxicity associated with cancer therapy (17, 18). Sunitinib, a multi-receptor TKI, is used as the first-line therapy for solid tumors including renal cell carcinoma, gastrointestinal stromal tumors, and pancreatic neuroendocrine tumors (19, 20). Despite its effectiveness, patients receiving sunitinib often experience both cardiac and vascular toxic effects (21, 22). The effects of sunitinib on vascular dysfunction are less well studied compared with its effect on cardiomyocyte toxicity (23, 24). Thus, elucidating the mechanisms underlying sunitinib-induced vascular toxicity is critical for mitigating its risk. Translational rewiring is implicated in cancer onset, progression, and resistance to anticancer therapies (25). Whether such a regulatory program functions in the context of vascular injury remains largely unknown.

Here we used human induced pluripotent stem cell–derived ECs (hiPSC-ECs) to study sunitinib-induced vascular toxicity. Via Ribo-Seq, we identified that sunitinib translationally inhibits staphylococcal nuclease and tudor domain–containing protein 1 (SND1) in vitro and in vivo. SND1, also known as p100 or tudor-SN, is a protein highly expressed in various cancers, including breast, liver, and colorectal cancers (26). SND1 is involved in multiple biological processes, including RNA splicing, transcription, RNA-induced silencing complex (RISC), and RNA epigenetics (27–29). As a highly conserved protein, SND1 contains a tandem repeat of 4 staphylococcal nuclease–like (SN-like) domains at the N-terminus and a tudor and SN domain at the C-terminus (30). Endothelial function and vascular integrity are critical modulators in cancer metastasis and progression (31, 32). Although much progress has been made in understanding SND1’s oncogenic role, the involvement of SND1 in endothelial function remains understudied.

Our study demonstrated that SND1 regulates endothelial function in vitro and in vivo. Through transcriptomic and functional studies, we found that SND1 transcriptionally regulated UBE2N, an E2-conjugating enzyme that mediates K63-linked ubiquitination. We found that knockdown (KD) of UBE2N exacerbated sunitinib-induced endothelial injury, while UBE2N overexpression (OE) rescued this injury by modulating recruitment of 53BP1, a critical regulator of the DNA damage response (DDR) pathway. This action of UBE2N requires the ubiquitin E3 ligases RNF8 and RNF168. Finally, we utilized in silico analysis of FDA-approved drugs and identified angiotensin-converting enzyme (ACE) inhibitors as a specific class of antihypertensive drugs that protect against sunitinib-induced vascular dysfunction both in vitro and in vivo, all the while preserving the efficacy of sunitinib against tumor growth. These protective effects were independent of ACE inhibitor BP control.

## Results

### Establishment of an hiPSC-EC model of sunitinib-induced endothelial dysfunction.

**Figure 1:**
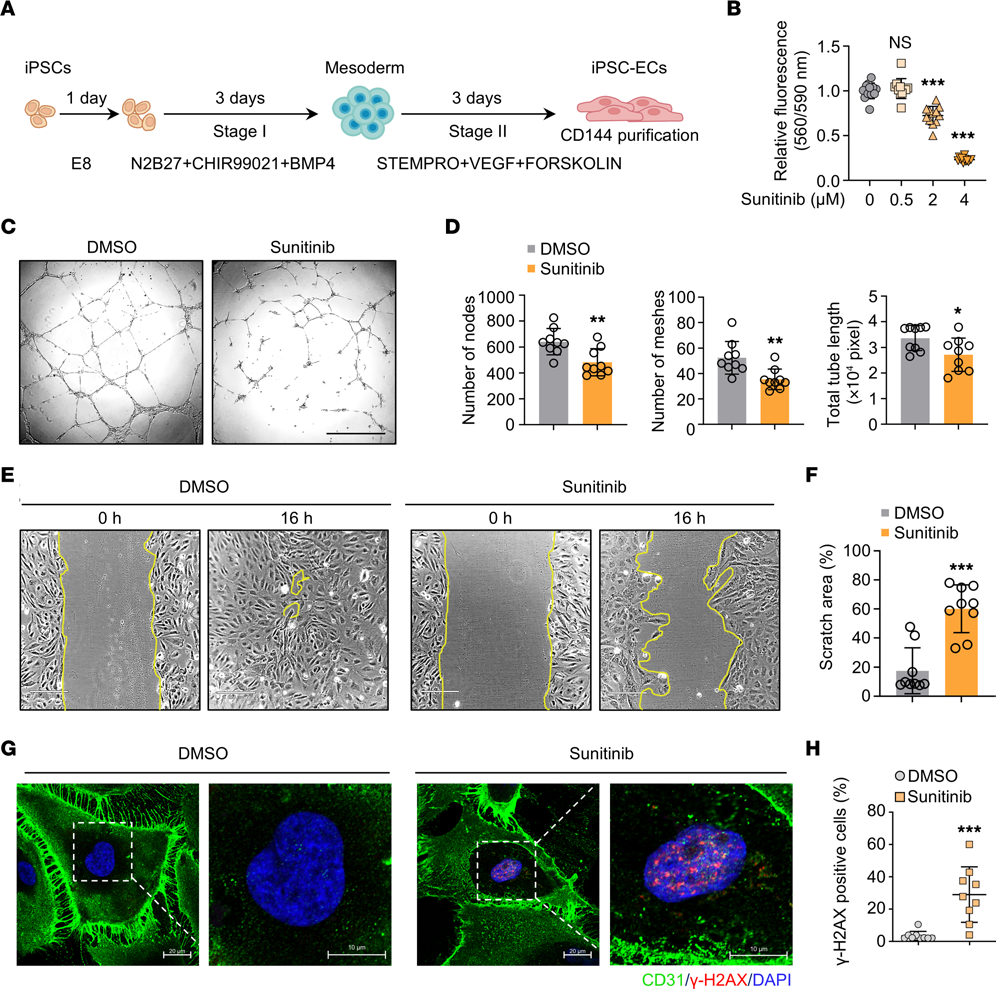
Generation of sunitinib-induced endothelial dysfunction model using hiPSCs. (**A**) Schematic representation of the hiPSC-to-EC (iPSC-EC) differentiation workflow. (**B**) hiPSC-ECs were treated with various concentrations of sunitinib for 48 hours. Cell viability was determined using the PrestoBlue cell viability reagent. One-way ANOVA. Data are presented as mean ± SD. ***P < 0.001; ns, not significant. *n* = 12 replicates from the differentiation of 3 individual hiPSC lines. (**C** and **D**) hiPSC-ECs were treated with 2 μM sunitinib for 48 hours. EC function was determined by tube-formation assay. *n* = 9 replicates from the differentiation of 3 individual hiPSC lines. Scale bar: 220 μm. Two-tailed Student’s *t* test. Data are presented as mean ± SD. *P < 0.05, **P < 0.01. (**E** and **F**) Representative images and quantification of wound-healing ability of hiPSC-ECs in response to treatment with DMSO or sunitinib (2 μM) for 16 hours. The yellow lines indicate the edges of the scratch wound. The scratched areas were quantified as a percentage relative to the initial area at 0 hours. *n* = 9 replicates from the differentiation of 3 individual hiPSC lines. Scale bars: 220 μm. Two-tailed Student’s *t* test. Data are presented as mean ± SD. ***P < 0.001. (**G** and **H**) Representative images of immunostaining of the DNA damage marker γ-H2AX (red) in hiPSC-ECs after sunitinib treatment (2 μM) for 48 hours. Cells were counterstained with CD31 (green), an EC marker. *n* = 9 replicates from the differentiation of 3 individual hiPSC lines. Scale bars: 20 and 10 μm. Two-tailed Student’s *t* test. Data are presented as mean ± SD. ***P < 0.001.

To study sunitinib-induced endothelial dysfunction in vitro, we differentiated 3 healthy hiPSC lines into ECs using a routine chemically defined protocol followed by CD144 purification (Figure 1A). Differentiated hiPSC-ECs highly expressed the EC-specific marker genes CD31 and CD144 (Supplemental Figure 1A; supplemental material available online with this article; https://doi.org/10.1172/JCI168730DS1). We subsequently treated hiPSC-ECs with sunitinib for 48 hours over a range of concentrations. We found that sunitinib significantly reduced viability in a dose-dependent manner, with 2 μM sunitinib causing approximately 30% cell death (Figure 1B), consistent with the occasional observation of capillary rarefaction in patients treated with sunitinib (22). We further confirmed the presence of sunitinib-induced endothelial dysfunction by assessing tube-formation ability and wound-healing rate in sunitinib-treated hiPSC-ECs compared with vehicle-treated controls (Figure 1, C–F). We observed that, consistent with findings in cancer cells (33), sunitinib also induced DNA damage in hiPSC-ECs, as indicated by an increase in the percentage of γ-H2AX–positive cells (Figure 1, G and H). Although sunitinib has been shown to induce hypertension clinically (34, 35), we did not observe any significant changes in phosphorylated eNOS upon sunitinib treatment in our model (Supplemental Figure 1, B and C). We next asked whether a lower dose of sunitinib (0.25 μM) can also impair endothelial function in both hiPSC-ECs and primary human ECs. We treated human aortic ECs (HAECs), HUVECs, and hiPSC-ECs with either 0.25 or 2 μM sunitinib. Although only a high dose of sunitinib (2 μM) reduced cell viability, both low and high doses of sunitinib led to dysfunction in hiPSC-ECs, HAECs, and HUVECs, as indicated by tube-formation and wound-healing assays (Supplemental Figure 1, D–L). To further confirm the cytotoxic effect of sunitinib on ECs, we measured glucose-6-phosphate dehydrogenase (G6PD), a cytosolic enzyme that is released when the plasma membrane integrity of cells is compromised. We observed that only a high concentration (2 μM) of sunitinib caused significant cytotoxicity in iPSC-ECs, whereas low concentrations (0.04 and 0.25 μM) did not cause cytotoxicity (Supplemental Figure 1M), results that were further confirmed by live/dead staining (Supplemental Figure 1N). We also tested the effect of 0.04 μM sunitinib on endothelial function. We confirmed that 0.04 μM sunitinib did not affect cell viability in hiPSC-ECs (Supplemental Figure 1O). Importantly, 0.04 μM sunitinib led to dysfunction in hiPSC-ECs, as indicated by inhibited angiogenesis (Supplemental Figure 1P). We also observed that 0.04 μM sunitinib treatment induced DNA damage in hiPSC-ECs (Supplemental Figure 1Q). These data strongly suggest that sunitinib treatment at a wide range of concentrations induces endothelial dysfunction, which can be recapitulated in hiPSC-ECs and primary human ECs.

### Ribo-Seq reveals that sunitinib translationally inhibits SND1.

**Figure 2:**
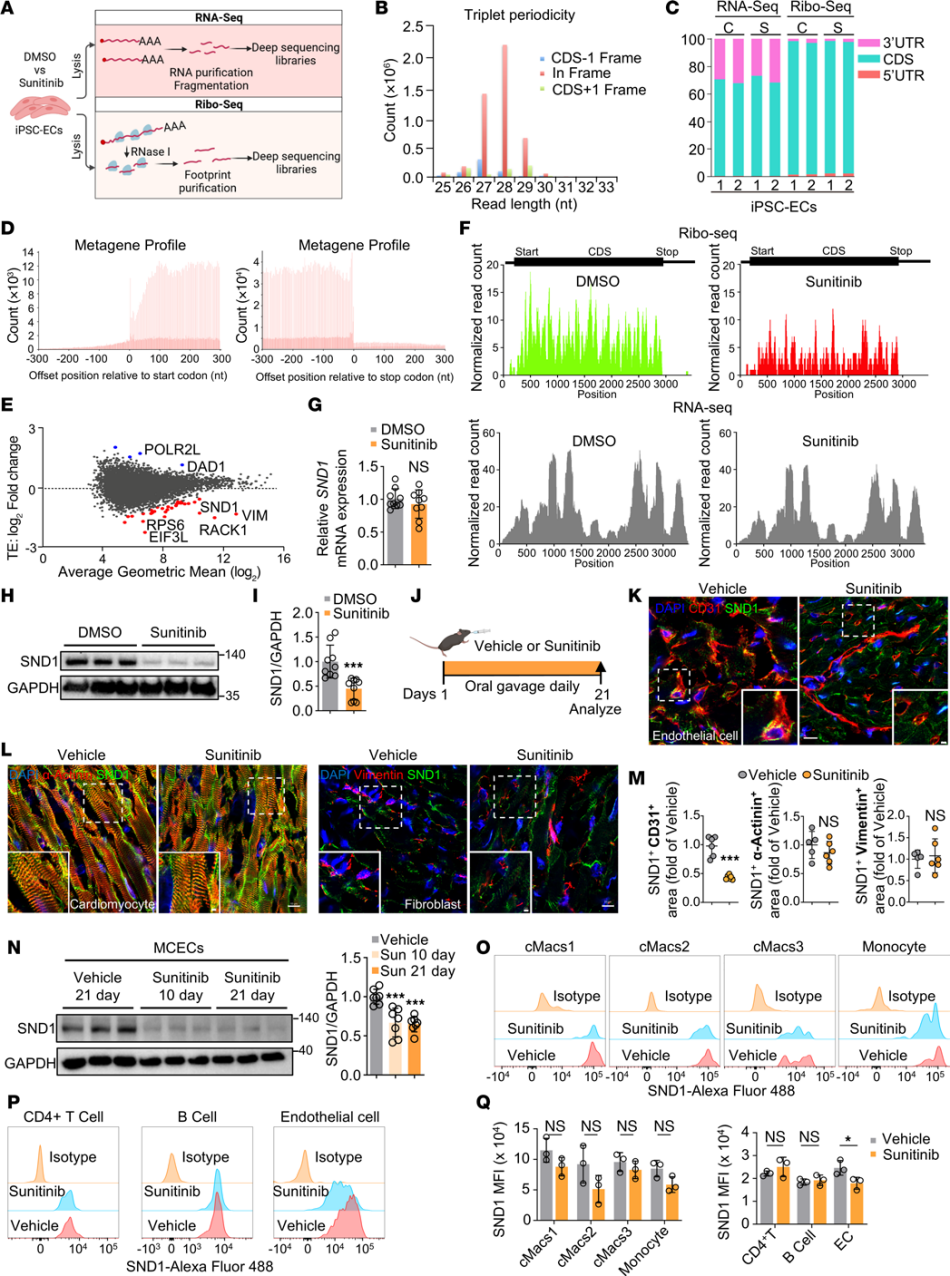
Ribo-Seq of hiPSC-ECs identified that sunitinib inhibits SND1 translation. (**A**) Schematic of RNA-Seq and Ribo-Seq of hiPSC-ECs to identify translationally regulated mRNAs in response to sunitinib treatment. Two independent hiPSC-EC lines were used for these analyses. (**B**) Read-length distribution and triplet periodicity for the Ribo-Seq dataset generated by RNase I. The reads are from a single principal transcript isoform from each gene. CDS, coding sequence. (**C**) Percentage of raw RNA-Seq and Ribo-Seq read counts over mRNA functional regions. C, control; S, sunitinib. (**D**) Metagene profile of normalized Ribo-Seq read density at the corresponding base positions relative to the start and stop codons. (**E**) Calculated translational efficiency (TE) of genes in sunitinib-treated (2 μM) versus DMSO-treated cells. Genes that did not change significantly are colored gray; upregulated genes are in blue; and downregulated genes are in red. (**F**) Normalized ribosome footprint (Ribo-Seq) reads and RNA-Seq reads of SND1 in response to DMSO or sunitinib treatment were analyzed by DESeq2. (**G**) RT-qPCR analysis of *SND1* expression in hiPSC-ECs following sunitinib treatment revealed that the mRNA level of SND1 was unaltered. Two-tailed Student’s *t* test. Data are presented as mean ± SD. *n* = 9 replicates from the differentiation of 3 individual hiPSC lines. (**H** and **I**) Immunoblot analysis and quantification of SND1 expression in hiPSC-ECs in response to DMSO or sunitinib treatment. Two-tailed Student’s *t* test. Data are presented as mean ± SD. ***P < 0.001. *n* = 9 replicates from the differentiation of 3 individual hiPSC lines. (**J**) Schematic summarizing the strategy for induction of sunitinib-induced vascular dysfunction in mice. (**K**) Representative images of SND1 and CD31 coimmunostaining of heart sections from the mice described in **J**. Scale bars: 10 μm and 2 μm. (**L**) Representative images of SND1 and α-actinin or vimentin coimmunostaining of heart sections from mice described in **J**. Scale bars: 10 μm and 2 μm. (**M**) Quantification of the SND1^+^CD31^+^ (*n* = 6), SND1^+^ α-actinin^+^ (vehicle, *n* = 5; sunitinib, *n* = 6), and SND1^+^vimentin^+^ (vehicle, *n* = 5; sunitinib, *n* = 6) areas. Two-tailed Student’s *t* test. Data are presented as mean ± SD. ***P < 0.001. (**N**) Immunoblot analysis of isolated MCECs from the mice after sunitinib (Sun) (10 and 21 days) or vehicle (21 days) treatment. One-way ANOVA. Data are presented as mean ± SD. ***P < 0.001. *n* = 7. (**O**–**Q**) Flow cytometry analysis of SND1 expression in different cardiac macrophage subsets and cardiac monocytes, T cells, B cells, and ECs in hearts from sunitinib-treated (21 days) or vehicle-treated (21 days) mice. Two-tailed Student’s *t* test. Data are presented as mean ± SD. *P < 0.05. *n* = 3.

To understand the effect of sunitinib on endothelial translation, we performed RNA-Seq and Ribo-Seq on hiPSC-ECs treated with DMSO or sunitinib (2 μM) for 24 hours (Figure 2A). RNase I produced a footprints with their length distribution peaking at 28 nucleotides as expected for ribosome protected fragments (Figure 2B). The alignments of footprints to coding regions (CDS) exhibited triplet periodicity characteristic for ribosome profiling data (Figure 2B). Ribo-Seq reads were mainly mapped to the CDS, with a negligible presence in UTRs, while RNA-Seq reads were mapped to both CDS and UTRs (Figure 2C), confirming the quality of the Ribo-Seq data. Metagene analysis revealed that ribosome footprint coverage largely overlapped with the reading frame being translated (Figure 2D). In addition, we found a high correlation in the Ribo-Seq and RNA-Seq data between biological replicates (Supplemental Figure 2A). Having verified the quality of our data, we next focused on the translational response to sunitinib in hiPSC-ECs. Translational efficiency (TE) was calculated by dividing normalized ribosome footprint reads by the normalized RNA-Seq reads (Figure 2E). Interestingly, among the genes translationally repressed in response to sunitinib treatment, we observed 2 classes — those that are related to ribosomal proteins/translation factors (e.g., RACK1, RPS6, EIF3L) and those that are related to nonribosomal/translational proteins (e.g., SND1, VIM). mRNAs in the first group encoding ribosomal proteins and translation factors typically contain a 5′-terminal oligopyrimidine tract (TOP) motif, which is characterized by a cytosine residue directly adjacent to the cap, followed by a continuous stretch of 4–15 pyrimidine nucleotides (36). Given that these proteins are well-established targets of mTOR complex 1 (mTORC1) (37) and function as broad regulators of the translational machinery, we decided to focus on nonribosomal/translational proteins, as we hypothesize that the latter group may play a more specific role in endothelial function as opposed to proteins involved in regulating global translation. We decided to focus on *SND1*, a gene that has been shown to be pro-oncogenic (28, 38–40), with an uncharacterized role in endothelial biology. The Ribo-Seq profile of *SND1* demonstrated that footprint density significantly decreased across the *SND1* CDS in response to sunitinib treatment, indicating decreased translation (Figure 2F). Consistent with the ribosome footprinting data, RT-qPCR data showed that mRNA levels of SND1 were unaffected by sunitinib compared with control treatment (Figure 2G). In contrast, immunoblotting confirmed the reduction in SND1 protein levels in sunitinib-treated hiPSC-ECs compared with control cells, supporting the notion that SND1 is regulated by sunitinib at the translational but not transcriptional level (Figure 2, H and I). We also examined whether sunitinib affects de novo protein synthesis in hiPSC-ECs using the SUnSET method, which relies on incorporation of puromycin into nascent proteins (41). We observed that sunitinib exposure resulted in a global reduction in protein synthesis, as shown by a reduction in puromycin-labeled proteins (Supplemental Figure 2B).

We next sought to confirm the relevance of our findings in vivo. Using a previously reported mouse model of sunitinib-induced vascular dysfunction (Figure 2J), we treated C57BL/6 mice with vehicle or sunitinib (40 mg/kg/d) for 3 weeks (21). To investigate whether sunitinib affects SND1 expression in ECs and other major cell types within the heart, we performed coimmunostaining of SND1 with the EC marker CD31 (Figure 2K), the cardiomyocyte marker α-actinin, and the fibroblast marker vimentin (Figure 2L) in both vehicle- and sunitinib-treated mouse hearts. SND1 highly colocalized with CD31 in vehicle-treated hearts, and this colocalization was significantly reduced in the sunitinib-treated group (Figure 2, K and M). In cardiomyocytes and fibroblasts, there was no discernible difference in SND1 expression between the control and sunitinib groups (Figure 2, L and M). Next, we isolated mouse cardiac ECs (MCECs) from vehicle- or sunitinib-treated mice using CD31-conjugated Dynabeads as previously reported (42). Close to 95% of the isolated cells were CD31-positive (Supplemental Figure 2, C and D), and these cells expressed SND1 as expected (Supplemental Figure 2E). We observed that, consistent with our immunostaining data, MCECs from sunitinib-treated mice had an approximately 40% reduction in the levels of SND1 protein as early as 10 days after treatment, and this reduction was sustained at 3 weeks as measured by immunoblotting (Figure 2N). Immunohistochemical staining also confirmed that sunitinib promoted downregulation of SND1 in the aortic lumen (Supplemental Figure 2F). Additionally, we treated hiPSC-derived cardiomyocytes (hiPSC-CMs) and human primary cardiac fibroblasts (HCFs) with various concentrations of sunitinib. Immunoblotting demonstrated that sunitinib did not affect SND1 expression in hiPSC-CMs and HCFs (Supplemental Figure 2, G and H), which aligns with our in vivo data. Together, these data demonstrate that sunitinib-mediated translational repression of SND1 is an EC-specific phenomenon.

As SND1 has been reported to regulate the immune system (43), we tested whether sunitinib represses SND1 expression not only in cardiac ECs, but also in cardiac-resident macrophages, monocytes, CD4^+^ T cells, and CD19^+^ B cells within the hearts of sunitinib-treated mice. As previously reported (44), we defined cardiac-resident macrophages (cMacs) as CD45^+^CD11b^+^F4/80^+^ cells and further separated them into 3 distinct subsets based on MHC class II (MHCII) and Ly6C expression (Supplemental Figure 2I). Importantly, we observed that in hearts of sunitinib-treated mice, expression of SND1 was not significantly altered in any of the following populations: Ly6C-MHCII^hi^ (cMacs1), Ly6C-MHCII^lo^ (cMacs2), Ly6C^+^MHCII^+^ (cMacs3), or MHCII^NEG^ cardiac monocytes (Figure 2, O and Q). Additionally, we evaluated SND1 expression in CD4^+^ T cells, CD19^+^ B cells, and CD31^+^ ECs from sunitinib-treated mouse hearts (Supplemental Figure 2J). Expression of SND1 was unaltered in either CD4^+^ T cells or CD19^+^ B cells (Figure 2, P and Q). In CD31^+^ ECs, though, SND1 expression was notably reduced in the hearts of sunitinib-treated mice (Figure 2, P and Q), in line with our earlier findings. These results collectively establish that SND1 is translationally suppressed by sunitinib and that this occurs selectively in ECs.

### Sunitinib translationally inhibits SND1 through the mTORC1/4E-BP1 pathway.

**Figure 3:**
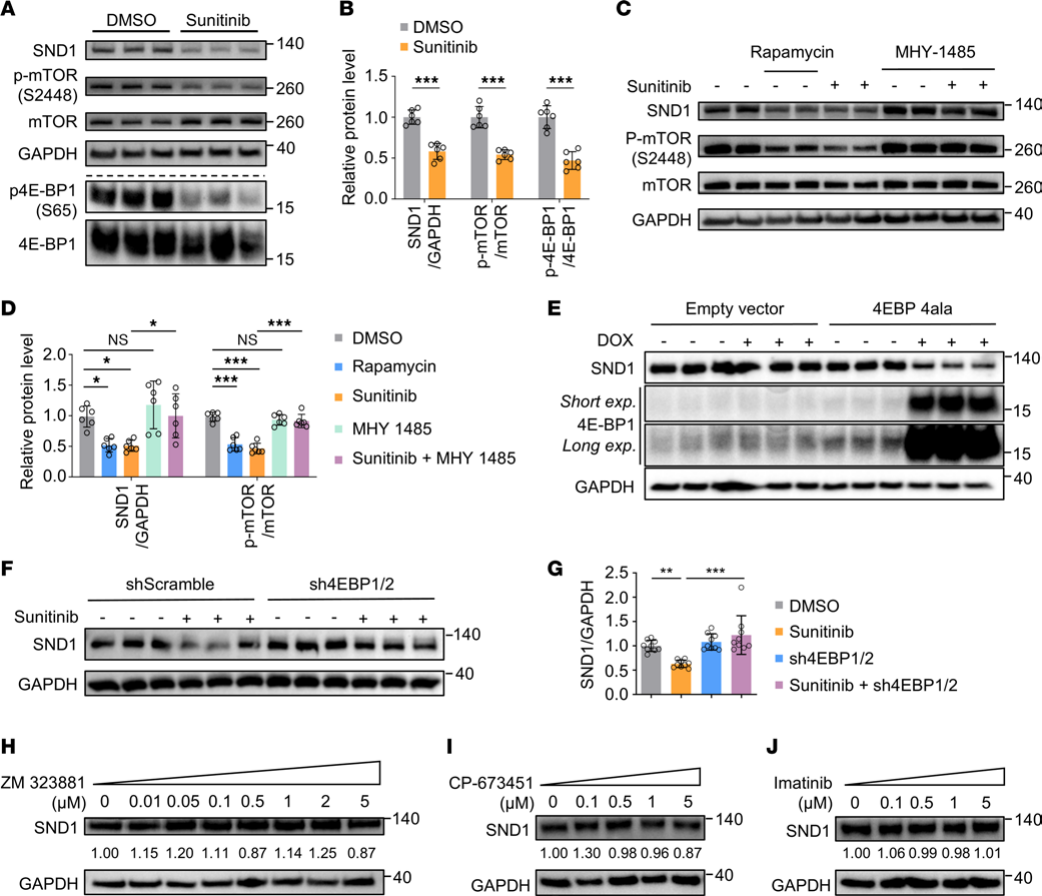
Inhibition of mTOR by sunitinib represses *SND1* translation via 4E-BP1. (**A** and **B**) Immunoblot analysis of SND1, mTOR, and its downstream target, 4E-BP1, in hiPSC-ECs treated with DMSO or sunitinib (2 μM) for 48 hours revealed a reduction in p-mTOR and p–4E-BP1 in response to sunitinib treatment. Two-tailed Student’s *t* test. Data are presented as mean ± SD. ***P < 0.001. *n* = 6 replicates from the differentiation of 2 individual hiPSC lines. (**C** and **D**) hiPSC-ECs were treated with rapamycin (100 nM) or sunitinib or pretreated with MHY-1485 (10 μM) for 12 hours before being treated with sunitinib (2 μM) or DMSO. Rapamycin treatment inhibited the SND1 level, and repression of SND1 by sunitinib was rescued by MHY-1485. One-way ANOVA. Data are presented as mean ± SD. *P < 0.05, ***P < 0.001. *n* = 6 replicates from the differentiation of 3 individual hiPSC lines. (**E**) hiPSC-ECs were transduced with lentivirus carrying a doxycycline-inducible (DOX-inducible) 4E-BP1-4Ala mutant gene. Following exposure (exp.) to DOX (1 μg/mL) for 48 hours, protein expression of SND1 and 4E-BP1 was detected by immunoblotting. (**F** and **G**) hiPSC-ECs were transduced with shScramble (shScr) or shRNAs against 4E-BP1 and 4E-BP2 (sh4E-BP1/2), before being treated with sunitinib (2 μM) or DMSO. Repression of SND1 by sunitinib was rescued when both 4E-BPs were genetically suppressed. One-way ANOVA. Data are presented as mean ± SD. **P < 0.01, ***P < 0.001. *n* = 9 replicates from the differentiation of 3 individual hiPSC lines. (**H**) hiPSC-ECs were treated with ZM 323881 (VEGFR2 inhibitor) at different concentrations for 24 hours. Immunoblot analysis indicated that ZM 323881 did not affect SND1 in ECs. (**I**) hiPSC-ECs were treated with CP-673451 (PDGFR inhibitor) at different concentrations for 24 hours. Immunoblot analysis indicated that CP-673451 did not affect SND1 in ECs. (**J**) hiPSC-ECs were treated with imatinib (c-Kit and PDGFR inhibitor) at different concentrations for 24 hours. Numerical values below the blots indicate quantification of SND1 bands relative to GAPDH. Immunoblot analysis indicated that imatinib did not affect SND1 in ECs.

Activation of mTORC1 phosphorylates the translation repressor eukaryotic translation initiation factor 4E–binding proteins (4E-BPs), releasing them from eukaryotic translation initiation factor 4E (eIF4E) to allow cap-dependent translation to proceed (45, 46). Previous studies demonstrated that sunitinib inhibits mTORC1 activity in a dose-dependent manner (47). We next investigated whether sunitinib-mediated inhibition of mTORC1 was involved in the translational inhibition of SND1 via 4E-BP1. We first determined the activation status of mTOR and 4E-BP1 in sunitinib-treated hiPSC-ECs. Immunoblotting revealed that phosphorylation of both mTOR and 4E-BP1 was significantly reduced following sunitinib treatment (Figure 3, A and B). To examine whether inhibited mTORC1 signaling was involved in repressing *SND1* translation, we treated hiPSC-ECs with 100 nM rapamycin (48) (an allosteric mTOR inhibitor) or 10 μM MHY-1485 (49) (a potent mTOR activator) in the presence of sunitinib. As expected (50), rapamycin significantly repressed mTOR autophosphorylation. Importantly, rapamycin-mediated inhibition of mTOR was accompanied by reduced SND1 expression, phenocopying the effects of sunitinib (Figure 3, C and D). Notably, sunitinib-induced inhibition of SND1 was partially relieved when cells were cotreated with MHY-1485, with a corresponding increase in the phosphorylation of mTOR (Figure 3, C and D). To further establish that 4E-BPs mediate the sunitinib-dependent translational inhibition of SND1 downstream of mTORC1, we transduced hiPSC-ECs with a doxycycline-inducible lentiviral construct overexpressing 4E-BP1-4Ala (37) (a non-phosphorylatable mutant of 4E-BP1 in which all 4 mTORC1-sensitive phosphorylation sites are mutated to Ala, resulting in constitutive 4E-BP1 binding to eIF4E) or control vector. In support of our hypothesis, exposure to doxycycline for 48 hours decreased SND1 levels compared with the control (Figure 3E). To further confirm the regulation of SND1 by 4E-BPs, we also transduced hiPSC-ECs with either scramble shRNA or shRNA targeting 4E-BP1/2, followed by sunitinib treatment. Immunoblotting indicated that the repression of SND1 by sunitinib was abolished when both 4E-BP1 and 4E-BP2 were suppressed (Figure 3, F and G). In addition to 4E-BPs, eIF4E activity is regulated by phosphorylation, which is mediated by MAPK-interacting kinases (MNKs) (51). However, treatment of hiPSC-ECs with eFT-508 (52), a selective inhibitor of MNK1/2, did not affect SND1 levels (Supplemental Figure 3A), further implicating the mTOR/4E-BP1 axis as the primary pathway mediating sunitinib-dependent translational repression of SND1. Both low and high doses of sunitinib could inhibit SND1 levels in multiple EC types (Supplemental Figure 3, B and C).

Sunitinib is a multitargeted TKI that targets more than 60 receptor tyrosine kinases, including VEGFR2, PDGFRβ, and c-KIT (53). We thus tested whether inhibition of these receptors mediates the suppressive effects of sunitinib on SND1 by treating hiPSC-ECs with ZM 323881 (VEGFR2 inhibitor), CP-673451 (PDGFR inhibitor), and imatinib (c-KIT and PDGFR inhibitor). We first verified that sunitinib inhibited VEGF165-induced VEGFR-2 phosphorylation in a concentration-dependent manner in hiPSC-ECs exposed to VEGF165 (Supplemental Figure 3D). We then observed that treatment of hiPSC-ECs with ZM 323881, CP-673451, or imatinib across an increasing range of concentrations did not have any discernible effects on SND1 protein levels (Figure 3, H–J), suggesting that the inhibitory effects of sunitinib on SND1 appear to be independent of the targeting of these RTKs. The PI3K/AKT/mTOR signaling pathway plays a pivotal role in regulating growth in both normal and cancer cells (54). Activation of PI3K phosphorylates and activates AKT, which in turn triggers activation of mTOR. We conducted a more in-depth analysis of the effect of the PI3K/AKT/mTOR axis on SND1 expression. We treated hiPSC-ECs with a PI3Kα inhibitor, BYL-719, or an AKT inhibitor, MK2206. As expected, treatment with both BYL-719 and MK2206 resulted in reduced PI3K/AKT/mTOR signaling, along with a marked reduction in SND1 protein levels (Supplemental Figure 3, E and F). Crucially, we found that ZM 323881 did not affect phospho-AKT and phospho-mTOR in hiPSC-ECs (Supplemental Figure 3G), further confirming our aforementioned results suggesting that sunitinib-mediated mTOR/4E-BP1 modulation of SND1 is independent of VEGFR2 inhibition.

### SND1 is a critical regulator of endothelial health.

**Figure 4:**
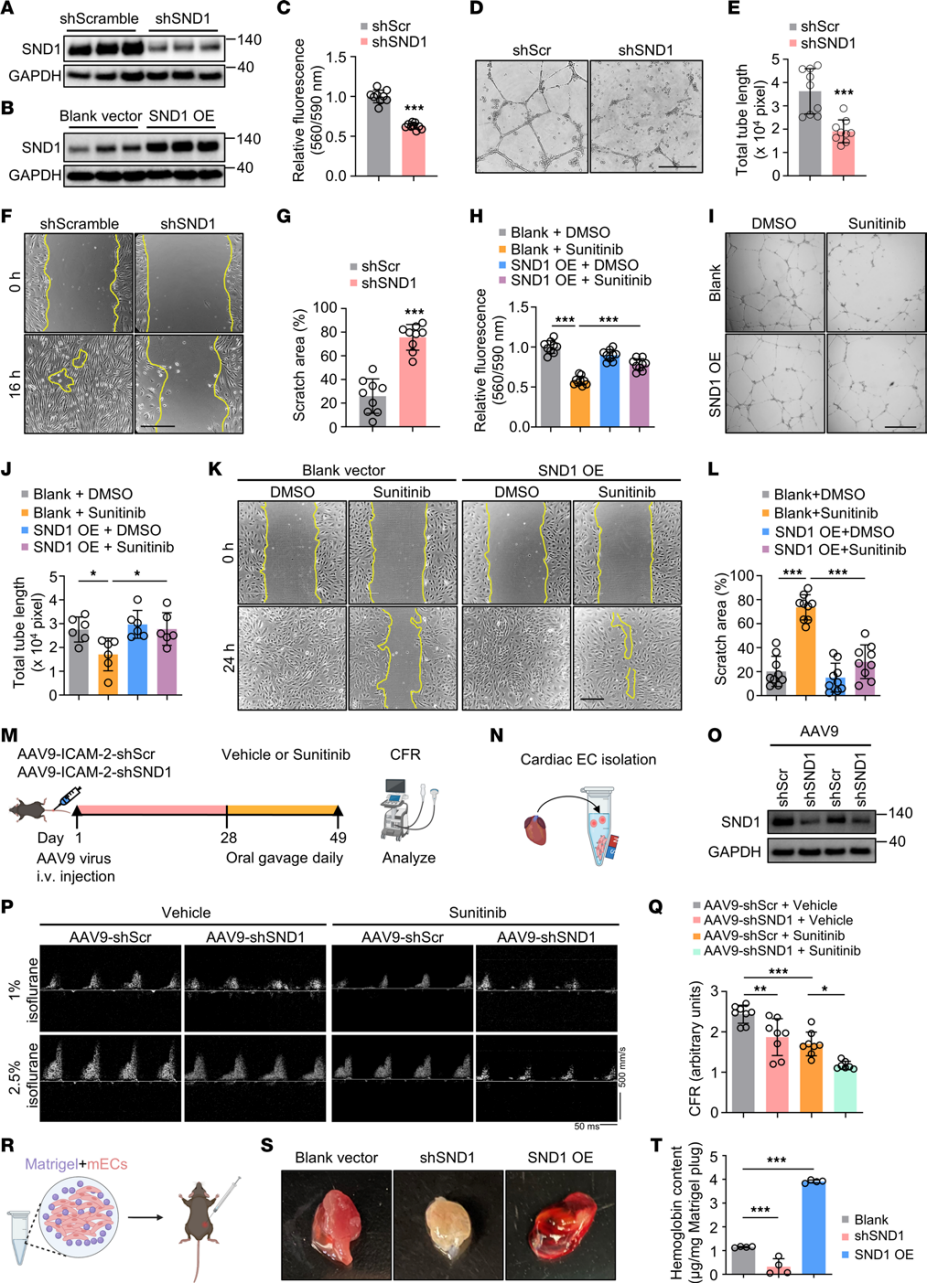
SND1 is a critical regulator of endothelial health. (**A** and **B**) The efficacy of SND1 KD and SND1 OE in hiPSC-ECs was validated by immunoblotting. (**C**) The effects of SND1 KD on EC function were determined by PrestoBlue viability assay. Two-tailed Student’s *t* test. Data are presented as mean ± SD. ***P < 0.001. *n* = 9 replicates from the differentiation of 3 individual hiPSC lines. (**D** and **E**) Representative images and quantification of the tube-formation efficiency of hiPSC-ECs following KD of SND1. Scale bar: 340 μm. Two-tailed Student’s *t* test. Data are presented as mean ± SD. ***P < 0.001. *n* = 9 replicates from the differentiation of 3 individual hiPSC lines. (**F** and **G**) Representative images and quantification of the wound-healing ability of hiPSC-ECs following KD of SND1. Two-tailed Student’s *t* test. Data are presented as mean ± SD. ***P < 0.001. *n* = 9 replicates from the differentiation of 3 individual hiPSC lines. Scale bar: 220 μm. (**H**) PrestoBlue viability assay in sunitinib-treated SND1 OE hiPSC-ECs. One-way ANOVA. Data are presented as mean ± SD. ***P < 0.001. *n* = 9 replicates from the differentiation of 3 individual hiPSC lines.(**I** and **J**) Effects of SND1 OE on hiPSC-ECs treated with DMSO or sunitinib (2 μM) were determined by tube-formation assay. One-way ANOVA. Data are presented as mean ± SD. *P < 0.05. *n* = 6 replicates from the differentiation of 2 individual hiPSC lines. Scale bar: 220 μm. (**K** and **L**) Wound-healing assay on sunitinib-treated SND1 OE hiPSC-ECs. One-way ANOVA. Data are presented as mean ± SD. ***P < 0.001. *n* = 9 replicates from the differentiation of 3 individual hiPSC lines. Scale bar: 220 μm (**M**) Schematic of experimental design to assess the physiological significance of SND1 in vivo. (**N** and **O**) Immunoblot analysis revealed that AAV9-shSND1 treatment inhibited SND1 protein levels in MCECs. (**P**) Representative ultrasound tracings of dilated (induced with 2.5% isoflurane) and basal (with 1% isoflurane) coronary flow. (**Q**) Quantification of CFR (dilated/basal flow) in mice. One-way ANOVA. Data are presented as mean ± SD. *P < 0.05, **P < 0.01, ***P < 0.001. AAV9-shSND1 + Sunitinib: *n* = 7. Other groups: *n* = 8 each. (**R**) Schematic of the Matrigel plug assay. mECs, mouse ECs labeled with CM-Dil dye. (**S** and **T**)) Representative gross images and hemoglobin content of explanted Matrigel plugs. One-way ANOVA. Data are presented as mean ± SD. ***P < 0.001. *n* = 4.

Having shown that SND1 is translationally repressed by sunitinib, we next assessed whether SND1 is a factor critical for endothelial function using gain- and loss-of-function studies. We first evaluated the effects of SND1 KD on EC function. Successful shRNA-mediated SND1 KD in hiPSC-ECs was confirmed by immunoblotting (Figure 4A). We found that SND1 deficiency in hiPSC-ECs resulted in reduced viability, impaired tube formation, and delayed wound healing, implicating SND1 as a pivotal regulator of endothelial function (Figure 4, C–G). These results were further verified in primary HAECs and HUVECs, which also demonstrated impaired endothelial function upon the loss of SND1 (Supplemental Figure 4, A–F). Of note, SND1 KD did not affect the viability of hiPSC-CMs and HCFs (Supplemental Figure 4, G and H), suggesting the specificity of SND1 as a unique regulator of endothelial function. Since SND1 deficiency leads to endothelial dysfunction, we reasoned that SND1 OE may counteract sunitinib-induced endothelial dysfunction. In line with our hypothesis, SND1 OE, which was confirmed by immunoblotting (Figure 4B), successfully reversed the detrimental effects of sunitinib on hiPSC-ECs (Figure 4, H–L), further supporting the role of SND1 in mediating endothelial homeostasis.

To assess the physiological significance of SND1 in vivo, we employed EC-specific adeno-associated virus serotype 9 (AAV9) carrying intercellular adhesion molecule 2 (ICAM-2) promoter driving SND1-shRNA (AAV9-shSND1) or scramble shRNA (AAV9-shScr). Mice were injected with AAV9-shScr or AAV9-shSND1 and, after 4 weeks of transduction, orally administered sunitinib (40 mg/kg/d) or vehicle for 3 weeks (Figure 4M). Immunoblotting of isolated MCECs confirmed the successful KD of SND1 in the AAV9-shSND1 compared with the vehicle control group (Figure 4, N and O). After 3 weeks of drug treatment, we measured coronary flow reserve (CFR) in the mice, quantified as the ratio of hyperemic to resting myocardial blood flow, which is an index of coronary microvascular function. Previous studies have shown that reduced CFR is associated with major adverse cardiovascular events (55). As expected, AAV9-shScr plus sunitinib treatment significantly reduced CFR compared with AAV9-shScr plus vehicle treatment, indicating impaired vascular function (Figure 4, P and Q). Importantly, SND1 KD not only impaired vascular function but also exacerbated sunitinib-induced vascular dysfunction (Figure 4, P and Q). In addition, we utilized an in vivo angiogenesis model by implanting a Matrigel plug supplemented with control, SND1 KD, or SND1 OE mouse cardiac ECs that were labeled with CM-Dil dye in the flank region of C57BL/6 mice (Figure 4R). The plugs were excised, photographed, and embedded in paraffin, and sections were stained for CD31 1 week after implantation. Interestingly, SND1 OE strongly induced blood vessel formation, which indicated the development of a functional vasculature (Figure 4S). On the other hand, plugs supplemented with SND1-KD ECs showed only minimal blood vessel formation (Figure 4S). The extent of plug vascularization was also evaluated by measuring hemoglobin (Hb) content; consistent with the macroscopic appearance, the plugs containing SND1-OE cells had the highest Hb content, followed by those with control cells, while SND1 KD dramatically suppressed the Hb content of the plugs (Figure 4T). Immunostaining of these Matrigel sections also confirmed that levels of CD31/CM-DiI double-positive cells were significantly reduced upon SND1 KD but upregulated upon SND1 OE (Supplemental Figure 4I). Taken together, these results indicate the essential role of SND1 in regulating endothelial function and angiogenesis both in vitro and in vivo.

### SND1 regulates endothelial homeostasis via UBE2N/RNF8/RNF168.

**Figure 5:**
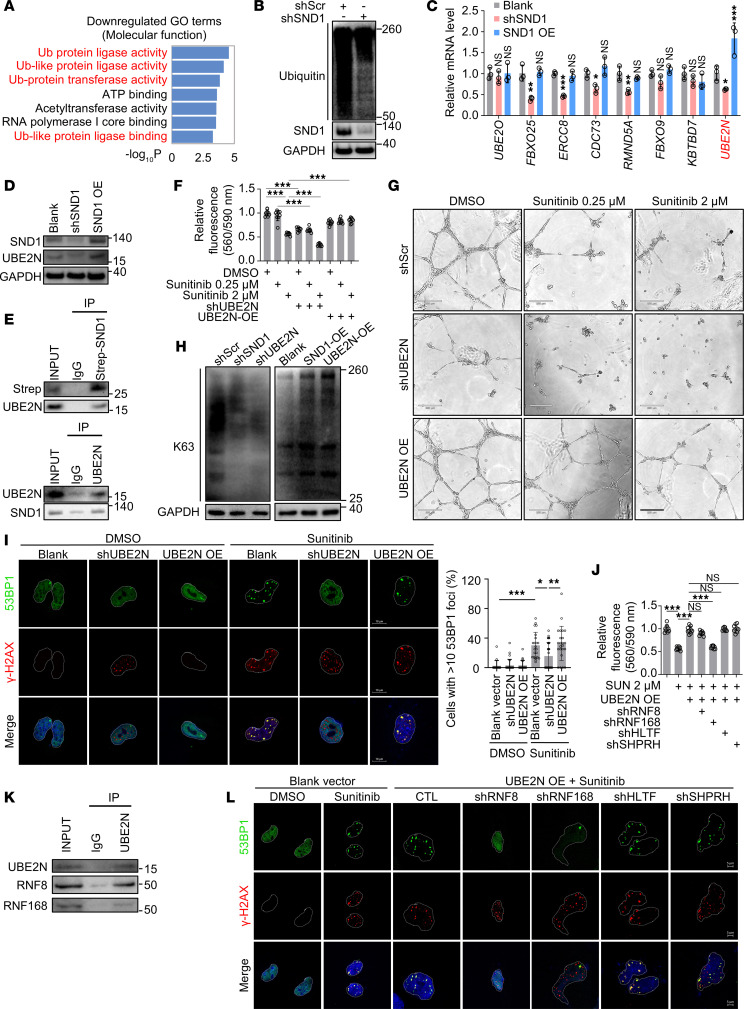
SND1 regulates endothelial homeostasis via UBE2N. (**A**) Gene ontology (GO) analysis of DEGs in RNA-Seq data obtained from hiPSC-ECs transduced with scramble shRNA or shSND1. Multiple ubiquitination-related processes were enriched among downregulated genes in SND1-KD hiPSC-ECs. (**B**) Immunoblot analysis confirmed that suppression of SND1 in hiPSC-ECs was associated with overall reduced levels of total ubiquitinated proteins. (**C**) RT-qPCR was performed on selected targets from RNA-Seq analysis to identify genes that were oppositely expressed in hiPSC-ECs after SND1 OE versus KD. *n* = 3 technical replicates. One-way ANOVA. Data are presented as mean ± SD. *P < 0.05, **P < 0.01, ***P < 0.001. (**D**) Immunoblotting demonstrating that UBE2N was reduced in shSND1 hiPSC-ECs and increased in SND1-OE hiPSC-ECs. (**E**) Co-IP analysis of the association between SND1 and UBE2N. (**F**) Effects of shUBE2N or UBE2N-OE on sunitinib-induced endothelial injury were determined by viability assay. One-way ANOVA. Data are presented as mean ± SD. ***P < 0.001. *n* = 9 replicates from the differentiation of 3 individual hiPSC lines. (**G**) Effects of shUBE2N or UBE2N OE on sunitinib-induced endothelial injury were determined by tube-formation assay. Scale bars: 220 μm. (**H**) Representative immunoblotting demonstrating that suppression of either SND1 or UBE2N led to downregulation of K63-linked polyubiquitination, while the overexpression of either SND1 or UBE2N led to upregulation of K63 in hiPSC-ECs. (**I**)Immunostaining of 53BP1 and γ-H2AX in hiPSC-ECs. DAPI was used for nuclear staining. Scale bars: 10 μm. One-way ANOVA. Data are presented as mean ± SD. *P < 0.05, **P < 0.01, ***P < 0.001. Blank+DMSO: 123 cells were quantified, *n* = 17 replicates. shUBE2N+DMSO: 151 cells were quantified, *n* = 26 replicates. UBE2N OE+DMSO: 143 cells were quantified, *n* = 19 replicates. Blank+sunitinib: 139 cells were quantified, *n* = 22 replicates. shUBE2N+sunitinib: 100 cells were quantified, *n* = 26 replicates. UBE2N OE+sunitinib: 131 cells were quantified, *n* = 23 replicates. (**J**) Cell viability assays showed that the protection conferred by UBE2N OE against sunitinib (SUN) was abrogated upon KD of RNF168 but not RNF8, HLTF, or SHPRH. One-way ANOVA. Data are presented as mean ± SD. ***P < 0.001. *n* = 9 replicates from the differentiation of 3 individual hiPSC lines. (**K**) Co-IP analysis of the association between UBE2N and RNF8 or RNF168. (**L**) hiPSC-ECs were transduced with blank vector, UBE2N OE, shScramble (CTL), shRNF8, shRNF168, shHLTF, or shSHPRH and then treated with DMSO or sunitinib, followed by immunostaining against 53BP1 and γ-H2AX. DAPI was used for nuclear staining. Scale bars: 5 μm.

To elucidate the molecular basis for the regulation of endothelial function by SND1, we then performed RNA-Seq on control and SND1-KD hiPSC-ECs. Notably, SND1 inhibition altered the abundance of 162 genes (fold change >1.5, *P* < 0.05), of which 124 genes were upregulated and 38 were downregulated (Supplemental Figure 5A). Intriguingly, gene ontology analysis revealed that differentially expressed genes (DEGs) were enriched for multiple protein ubiquitination–related pathways (Figure 5A). Immunoblotting using a pan-ubiquitin antibody confirmed that SND1 inhibition led to a decrease in total ubiquitin levels in SND1-KD hiPSC-ECs, in support of our RNA-Seq data (Figure 5B). We next selected several DEGs that are known to be involved in ubiquitination-related pathways and were found to be downregulated in the RNA-Seq data for SND1-KD hiPSC-ECs. RT-qPCR was performed to assess expression of these genes in response to SND1 KD and OE in hiPSC-ECs. Our analysis identified UBE2N (also known as UBC13), an E2 ubiquitin-conjugating enzyme (56), as an SND1-sensitive candidate gene (Figure 5C). Immunoblotting confirmed that the UBE2N level was downregulated upon SND1 KD but upregulated upon SND1 OE (Figure 5D). Additionally, sunitinib treatment of hiPSC-ECs led to repression of UBE2N, an effect that was abrogated upon SND1 OE (Supplemental Figure 5B). SND1 can regulate expression and stability of other proteins through direct protein-protein interactions (38, 39). We next assessed whether SND1 and UBE2N physically interact. We performed coimmunoprecipitation assay upon OE of a Strep-tagged SND1 construct in hiPSC-ECs. Immunoprecipitation with anti-Strep antibody followed by immunoblotting with anti-UBE2N antibody confirmed the interaction of SND1 and UBE2N (Figure 5E). Reciprocal pulldown of endogenous UBE2N further established its interaction with SND1 (Figure 5E).

Having shown that SND1 regulates expression of UBE2N in ECs, we tested the notion that UBE2N acts downstream of SND1 and is implicated in sunitinib-induced endothelial dysfunction. We transduced hiPSC-ECs with shRNA targeting UBE2N or UBE2N-OE lentivirus, and then treated the cells with sunitinib. Consistent with UBE2N acting downstream of sunitinib, UBE2N KD not only impaired endothelial function but also exacerbated sunitinib-induced hiPSC-EC injury, effects that were rescued by UBE2N OE (Figure 5, F and G). We also confirmed the regulatory role of UBE2N in HAECs and HUVECs (Supplemental Figure 6, A–E). To further explore the role of UBE2N, we utilized a selective UBE2N inhibitor, NSC697923 (57). We confirmed that pharmacological inhibition of UBE2N induced hiPSC-EC death in a concentration-dependent manner (Supplemental Figure 7A). Induction of endothelial dysfunction in response to NSC697923 was further supported by impaired tube formation (Supplemental Figure 7, B and C). UBE2N is known to specifically generate K63-linked ubiquitin chains (56). We confirmed that downregulation of either UBE2N or SND1 resulted in reduced K63 ubiquitination, whereas OE of UBE2N or SND1 increased K63 ubiquitination (Figure 5H). Unlike conventional lysine 48–linked (K48-linked) polyubiquitination, which is involved in protein degradation, K63-linked ubiquitin chains are mainly involved in signaling cascades, including the DNA damage pathway (58). Hence, we asked whether impaired UBE2N-mediated DNA repair contributes to sunitinib-induced endothelial dysfunction in hiPSC-ECs. Previous studies have reported the relevance of UBE2N in forming K63-linked ubiquitin chains upon the occurance of DNA double-strand breaks (DSBs), which in turn promotes the recruitment of 53BP1, a factor that induces DNA repair through nonhomologous end-joining (NHEJ) (59, 60). Formation of both DSB biomarkers — γ-H2AX and 53BP1 foci— was observed in sunitinib-treated control (blank) and UBE2N OE cells; however despite the presence of γ-H2AX, the percentage of 53BP1 foci was significantly reduced in sunitinib-treated shUBE2N cells, indicating ineffective DNA repair (Figure 5I). Collectively, these findings strongly suggest that SND1 regulates endothelial homeostasis via UBE2N.

Cooperation between E2 ubiquitin-conjugating enzyme and E3 ubiquitin ligase is essential for ubiquitin processes (61). We next sought to identify which E3 ligase participates in the ubiquitination cascade along with UBE2N, which is essential for the DNA repair pathway in sunitinib-treated ECs. The E3 ligases ring finger protein 8 (RNF8) and RNF168 have previously been reported to interact with UBE2N upon DSB formation and facilitate DNA repair (62, 63). Likewise, helicase-like transcription factor (HLTF) and SNF2 histone linker PHD RING helicase (SHPRH) have also been implicated in UBE2N-mediated polyubiquitylation of proliferating cell nuclear antigen (PCNA), which in turn promotes error-free replication through DNA lesions (64). To identify the responsible E3 ligase, we inhibited each of these E3 ligases (RNF8, RNF168, HLTF, and SHPRH) concurrent with UBE2N OE in sunitinib-treated hiPSC-ECs. Notably, the protection conferred by UBE2N OE against sunitinib in terms of cell viability was abrogated upon knockdown of RNF168 but not RNF8, HLTF, or SHPRH (Figure 5J). Furthermore, wound-healing assays revealed that the protective effects of UBE2N OE on sunitinib-induced endothelial dysfunction were reversed by inhibition of RNF8 or RNF168 but not HLTF and SHPRH (Supplemental Figure 8). Coimmunoprecipitation of UBE2N revealed a physical interaction with both RNF8 and RNF168, further supporting the functional relationship between these E2 and E3 ligases (Figure 5K). We also investigated whether these E3 ligases regulate formation of 53BP1 foci after sunitinib exposure. Indeed, formation of 53BP1 foci in UBE2N-OE cells treated with sunitinib was inhibited by knocking down RNF8 or RNF168 but not HLTF or SHPRH (Figure 5L). Together, these results suggest that the SND1/UBE2N/RNF8-RNF168 axis is crucial in protecting against sunitinib-induced endothelial dysfunction by regulating the DDR pathway.

### Identification of small molecules that ameliorate sunitinib-induced endothelial dysfunction.

**Figure 6:**
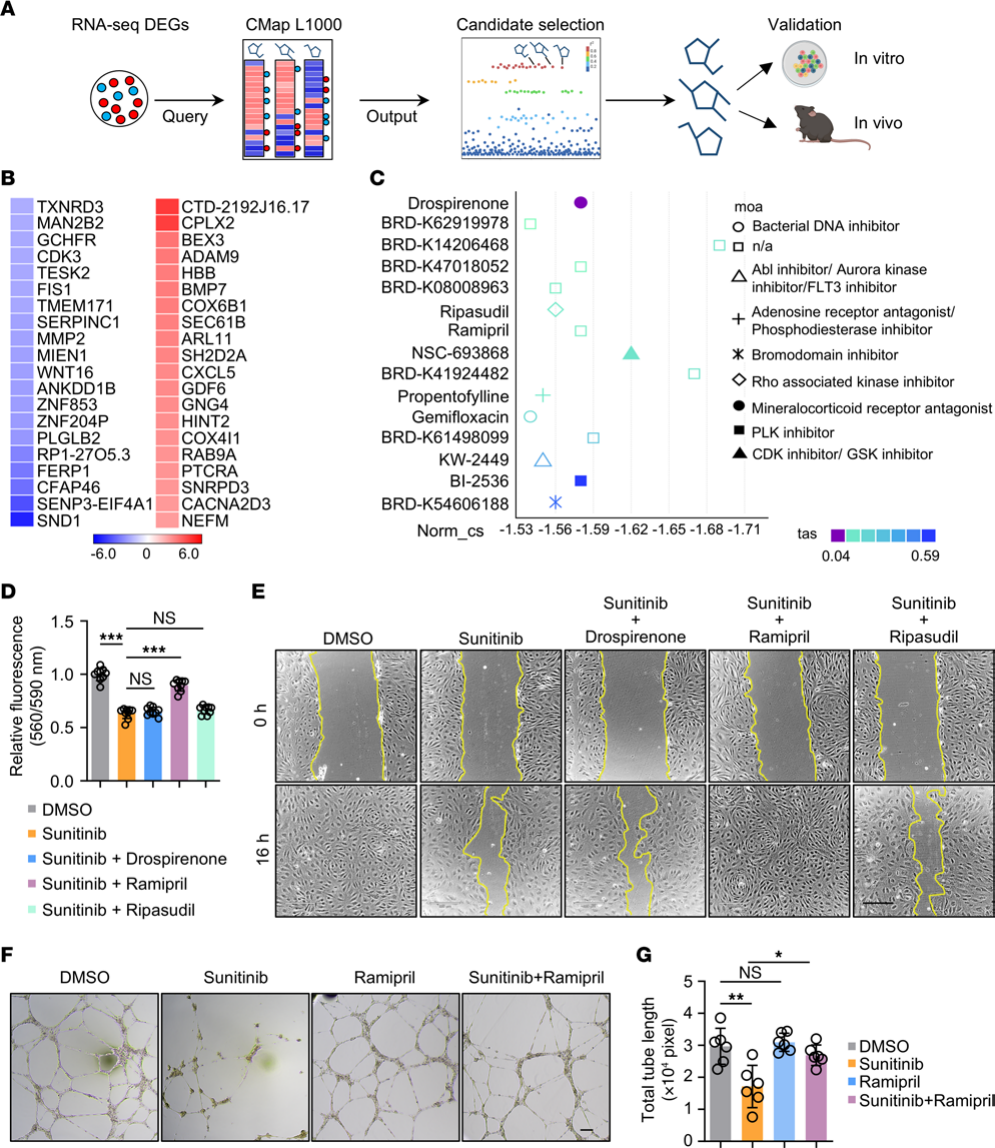
Identification of ramipril as a candidate compound that protects against sunitinib-induced vascular dysfunction. (**A**) Schematic of in silico drug screening using a Connectivity Map (CMap) approach. (**B**) Heatmap representing the shSND1-regulated genes (red: 20 most upregulated; blue: 20 most downregulated) obtained from RNA-Seq data. (**C**) Chemical compound ranking chart based on connectivity score (Norm_cs) and transcriptional activity score (tas). moa, mechanism of action. (**D**) hiPSC-ECs were treated with drospirenone (250 nM), ramipril (3 μM), or ripasudil (1 μM) along with sunitinib (2 μM) or DMSO for 48 hours. Cell viability was determined using the PrestoBlue cell viability reagent. One-way ANOVA. Data are presented as mean ± SD. ***P < 0.001. *n* = 9 replicates from the differentiation of 3 individual hiPSC lines. (**E**) Representative images of wound-healing ability of hiPSC-ECs treated with sunitinib (2 μM) for 48 hours in the presence and absence of drospirenone (250 nM), ramipril (3 μM), or ripasudil (1 μM). Treatment with DMSO was used as control. The yellow lines indicate the edges of the scratch wound. Scale bars: 220 μm. (**F** and **G**) hiPSC-ECs were treated with sunitinib (0.25 μM) for 48 hours in the presence and absence of ramipril (3 μM). Endothelial cell function was determined by tube-formation assay. Scale bar: 100 μm. One-way ANOVA. Data are presented as mean ± SD. *P < 0.05, **P < 0.01. *n* = 6 replicates from the differentiation of 3 individual hiPSC lines.

Our data thus far showed that SND1 OE preserved endothelial viability and function in the presence of sunitinib. However, since SND1 exerts oncogenic effects in selected cancers (38, 39, 65), treating patients with cancer by overexpressing SND1 may not be feasible. This is despite our analysis of the correlation between SND1 and cancer types represented in The Cancer Genome Atlas (TCGA) database indicating that SND1 is not elevated in kidney renal clear cell carcinoma (KIRC) and kidney renal papillary cell carcinoma (KIRP)—cancer types that sunitinib is commonly used to treat (Supplemental Figure 9). Nonetheless, to circumvent these potential concerns, we decided to focus on the direct effects of SND1 on ECs instead and hypothesized that pharmacological reversal of broad transcriptional changes caused by SND1 KD in ECs may protect against sunitinib-induced endothelial dysfunction. We took advantage of the Connectivity Map (CMap) database which contains gene expression profiles from several human cell lines in response to ~1,300 small molecule compounds (66). (1). An extension of the CMap project, L1000 (also known as LINCS-L1000 or CMap2), comprises gene expression profiles of approximately 50 human cell lines in response to ~20K compounds (67). We created a gene signature of SND1 KD hiPSC-ECs based on our aforementioned RNA-sequencing data (Figure 6, A and B) by selecting the top 150 upregulated genes and top 150 downregulated genes, ranked in order of fold-change with *P* < 0.05. Using this gene signature as input, we then queried the L1000 database for currently FDA-approved drugs that may reverse the signature of SND1 KD. Notably, among the class of candidate small molecules with the most significant connectivity score, drospirenone, ripasudil, and ramipril have been shown to play a role in endothelial biology (Figure 6C). Drospirenone (Slynd) is a progestin medication used to prevent pregnancy and in menopausal hormone therapy (68). Ripasudil (Glanatec) is a rho kinase inhibitor drug used to treat glaucoma and ocular hypertension (69). Ramipril (Altace) is an ACE inhibitor–type medication used to treat high blood pressure and heart failure (70). We selected these 3 candidate drugs and assessed whether they could counteract sunitinib-induced endothelial dysfunction by cotreating hiPSC-ECs with sunitinib along with each of them for 48 hours. Intriguingly, we found that of the 3 tested drugs, only ramipril, but not drospirenone or ripasudil, significantly alleviated the detrimental effects of sunitinib on hiPSC-ECs, as measured by cell viability, wound healing, and tube formation (Figure 6, D–G).

We next asked whether the protective effects of ramipril against sunitinib extend to other classes of antihypertensive drugs with distinct modes of action or are exclusive to ramipril’s role as an ACE inhibitor. We first evaluated 2 other classes of antihypertensive drugs, namely, atenolol (Tenormin), which acts as a beta blocker (71), and amlodipine (Norvasc), which acts as a calcium channel blocker (72). Interestingly, neither atenolol nor amlodipine showed protection against sunitinib-induced endothelial dysfunction (Supplemental Figure 10, A and B). Conversely, we found that 3 other commonly used ACE inhibitors, lisinopril, enalapril, and benazepril — which share the same basic chemical structure with ramipril (Supplemental Figure 11A) — were also protective against sunitinib, as indicated by wound-healing and tube-formation assays (Supplemental Figure 11, B and C). These results reveal a pivotal role for ACE inhibitors in counteracting the detrimental effects of sunitinib, although the exact mechanisms remain to be defined.

### Ramipril protects against sunitinib-induced vascular dysfunction while preserving its therapeutic efficacy against tumor in vivo.

**Figure 7:**
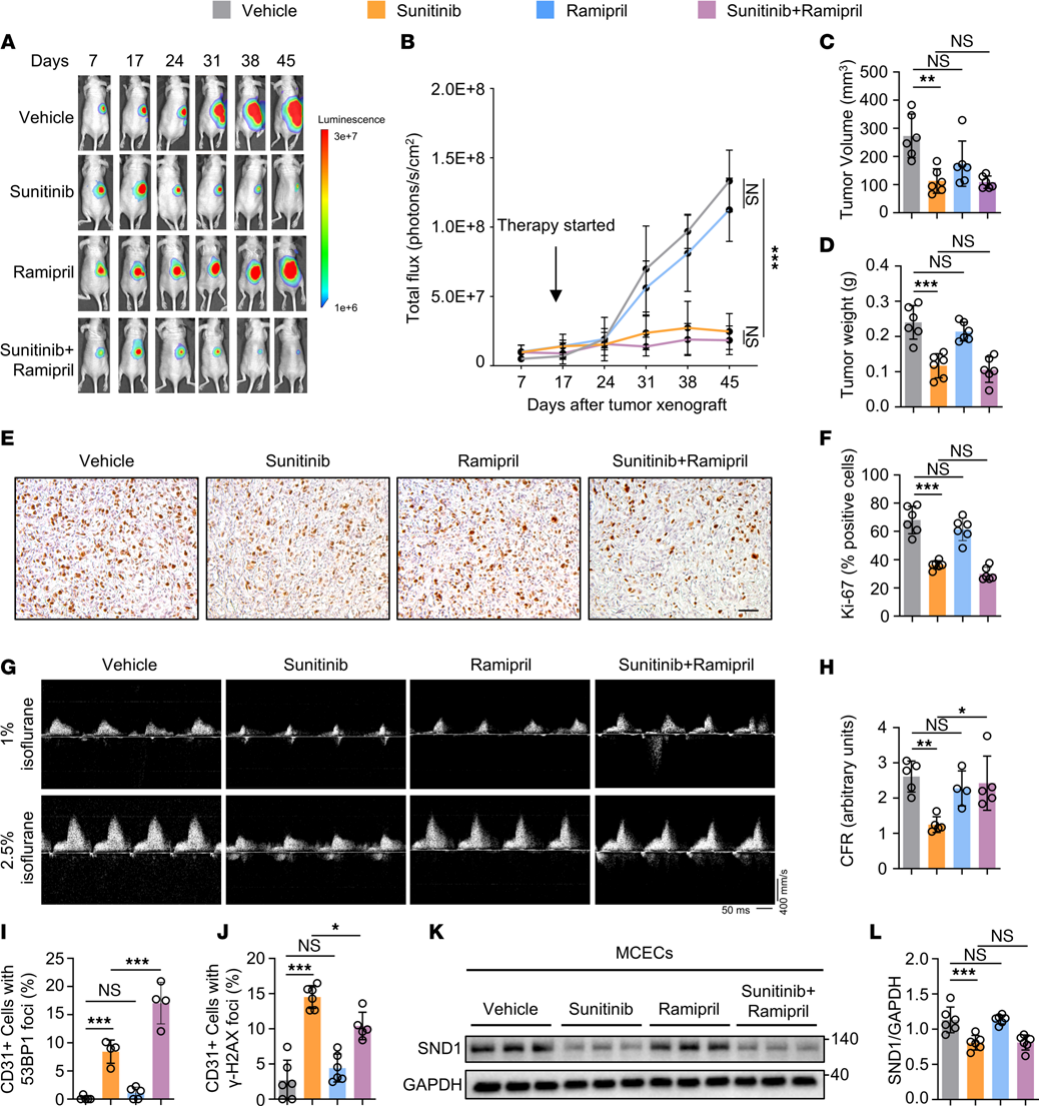
Ramipril alleviates sunitinib-induced vascular dysfunction without compromising its antitumor efficacy in vivo. Orthotopic tumors were induced via injection of 786-O-Fluc cells into right kidney (day 0). The indicated drugs or vehicle were administered via oral gavage once daily from day 16 to day 37. *n* = 6 mice per group. (**A**) Representative BLIs of nude mice. (**B**) Tumor growth was monitored weekly by BLI and measured as photons/s. Two-way ANOVA. Data are presented as mean ± SD. ***P < 0.001. *n* = 6 per group. Calculated tumor volume (**C**) and tumor weight (**D**) in each group on day 45 after tumor xenograft. *n* = 6 per group. One-way ANOVA. Data are presented as mean ± SD. **P < 0.01 ***P < 0.001. (**E** and **F**) Immunohistochemical staining of Ki-67 in tumors. Scale bar: 50 μm. One-way ANOVA. Data are presented as mean ± SD. ***P < 0.001. *n* = 6 in each group. (**G**) Representative ultrasound tracings of dilated (induced with 2.5% isoflurane) and basal (with 1% isoflurane) coronary flow after 21 days of treatment with sunitinib (40 mg/kg/d) in the presence and absence of ramipril (10 mg/kg/d). (**H**) Quantification of coronary flow reserve (CFR) (dilated/basal flow) in sunitinib-treated mice in the presence and absence of ramipril and corresponding vehicle-treated mice. One-way ANOVA. Data are presented as mean ± SD. *P < 0.05, **P < 0.01. Ramipril group: *n* = 4. Other groups: *n* = 5 each. (**I**) Quantification of CD31/53BP1 staining in heart sections of xenografted nude mice (day 45 after tumor xenograft). One-way ANOVA. Data are presented as mean ± SD. ***P < 0.001. Vehicle: *n* = 5. Sunitinib: *n* = 4. Ramipril: *n* = 5. Sunitinib+ramipril: *n* = 4. (**J**) Quantification of CD31/γ-H2AX staining in heart sections of xenografted nude mice (day 45 after tumor xenograft). One-way ANOVA. Data are presented as mean ± SD. *P < 0.05, ***P < 0.001. Sunitinib+ramipril group: *n* = 5. Other groups: *n* = 6 each. (**K** and **L**) Immunoblot analysis revealed that ramipril was unable to reverse sunitinib’s inhibition of SND1 in isolated MCECs (21 days after drug treatment). One-way ANOVA. Data are presented as mean ± SD. ***P < 0.001. *n* = 6.

To assess the prospective utility of ramipril as a cardiovascular protectant in the context of TKI therapy, we first evaluated the combinatorial effects of sunitinib and ramipril on tumor growth, as it is imperative that any potential candidate not adversely affect the therapeutic efficacy of sunitinib. Stable luciferase-expressing 786-O clear cell renal cell carcinoma (ccRCC) cells (786-O-Fluc) were orthotopically injected into immune-deficient (Nu/J) mice. Tumors were allowed to grow and reach a photon emission of approximately 10^7^ photons/s (2–3 weeks after xenograft) prior to initiation of daily oral administration of sunitinib alone (40 mg/kg/d), ramipril alone (10 mg/kg/d), sunitinib plus ramipril, or vehicle only and maintained for an additional period of 3 weeks. As anticipated, tumors displayed ongoing growth in mice treated with vehicle or ramipril alone, as evidenced by bioluminescence imaging (BLI) performed weekly (Figure 7, A and B). In contrast, tumors in mice treated solely with sunitinib exhibited clear regression. Tumors were also significantly reduced in mice treated with sunitinib combined with ramipril as compared with vehicle-treated mice, and the extent of the reduction was similar to that in mice receiving sunitinib alone (Figure 7, A and B). Tumor volume and weight data also supported this conclusion (Figure 7, C and D). Immunohistochemical staining of the proliferation marker Ki-67 confirmed that sunitinib plus ramipril were as effective as sunitinib alone in reducing proliferation when compared with vehicle (Figure 7, E and F). Collectively, these results indicate that the use of ramipril does not negate the effects of sunitinib in restraining RCC tumor growth. Having shown that ramipril does not affect the efficacy of sunitinib, we further tested whether ramipril is also cardioprotective in the tumor-xenografted mice receiving sunitinib. We measured CFR in these mice at the end of the experiments. As expected, sunitinib treatment significantly reduced CFR compared with vehicle treatment, indicating impaired vascular function. Importantly, in sunitinib-treated mice also receiving ramipril, CFR was restored (Figure 7, G and H). In addition, in mice treated with sunitinib alone or sunitinib and ramipril in combination, there were no significant differences in blood pressure compared with the control group (data not shown), indicating that the protective effects of ramipril against sunitinib were not due to its function in lowering blood pressure. Together, these results suggest that ramipril treatment protects against sunitinib-induced vascular dysfunction both in vitro and in vivo, all while preserving the efficacy of cancer therapy. We next explored whether ramipril mediates its cardioprotective effect via the SND1/UBE2N/DNA damage pathway. In line with a previous study linking ramipril to DNA damage repair after myocardial infarction (73), we observed that compared with sunitinib treatment, which promoted DNA damage, combined use of sunitinib and ramipril led to a significant decrease in γ-H2AX foci and a notable increase in 53BP1 foci in cardiac ECs (Figure 7, I and J); this suggested an increased DDR, which we showed to be regulated by SND1/UBE2N/RNF8/RNF168. These effects appear to be downstream of SND1, as ramipril was unable to reverse sunitinib’s inhibition of SND1 in isolated MCECs (Figure 7, K and L). These data suggests that ramipril can alleviate the DNA damage induced by sunitinib.

## Discussion

Control of mRNA translation is key for stress responses, as it allows specific cell types to respond rapidly to microenvironmental cues and stressors (25, 74). While the presence of translational regulatory networks has been extensively documented in cancer cells (75, 76), much less is known about translational regulation of the endothelium. The use of cancer drugs has been linked to vascular dysfunction (77). Whether this is associated with perturbed translational control remains unknown. Here we used hiPSCs-ECs to study the role of translational regulation in sunitinib-induced endothelial dysfunction. The major findings of this study are as follows: (i) Ribo-Seq revealed that sunitinib translationally inhibited *SND1*; (ii) sunitinib selectively inhibited SND1 in cardiac ECs; (iii) sunitinib-mediated mTOR inhibition suppressed *SND1* translation via 4E-BP1; (iv) in vitro and in vivo studies revealed SND1 as a regulator of endothelial function; (v) SND1 modulated endothelial homeostasis via the UBE2N/RNF8/RNF168/DNA damage pathway; and (vi) repurposing of FDA-approved drugs identified ACE inhibitors as a potential class of drugs that could prevent sunitinib-induced endothelial dysfunction, all the while preserving the efficacy of sunitinib in cancer therapy.

Compared with that of transcriptional regulation, our knowledge of translational regulation in the heart, especially pertaining to ECs, is still in its infancy (78). Doroudgar and colleagues identified that translational control determines early stages of gene expression alterations in cardiomyocytes in response to cardiac stress (4). A separate study provided evidence that translational control downstream of the PI3K/AKT and MAPK signaling pathways plays an important role in cardiomyocyte hypertrophy (79). Translational control is also reported to mediate the fibroblast-to-myofibroblast transition in the heart (80). Our study adds to this growing area of knowledge by linking translational control and cancer drug–induced vascular toxicity. Our translatome analysis of sunitinib-treated hiPSC-ECs identified *SND1* as a translationally regulated gene. We found that sunitinib inhibited *SND1* translation at a wide range of concentrations independent of cell death. Sunitinib repressed translation of SND1 via the mTOR/4E-BP1 pathway. Intriguingly, our data demonstrated that sunitinib-mediated inhibition of SND1 was EC-specific, as SND1 levels were not affected in other cell types within the heart. However, how SND1 is specifically regulated by mTOR/4E-BP1 remains unclear, and future studies are required to better understand this regulation.

As an evolutionarily conserved protein, SND1 has emerged not only as a biomarker (65, 81), but also as a promoter of cancer progression (82, 83). A previous study revealed that SND1 promotes tumor angiogenesis in human hepatocellular carcinoma (84), suggesting its involvement in modulating tumor vasculature and cancer progression. However, whether SND1 regulates endothelial function and vascular homeostasis under physiological conditions remains unknown. Results of our gain- and loss-of-function studies in vitro and in vivo demonstrate that translational control of SND1 played a critical role in regulating endothelial function. A recent study showed that sunitinib induces DNA damage and autophagy in the 786-O RCC cell line (33). Moreover, SND1 is required to repair DNA damage in mouse embryonic fibroblasts (85). However, whether and how the SND1/DDR pathway is involved in sunitinib-induced endothelial dysfunction remains enigmatic. Our study suggests that SND1 is essential for DNA repair in ECs and that the E2 enzyme UBE2N acts as a linker between SND1 and DNA damage. To the best of our knowledge, the contribution of E2 enzyme in TKI-induced vascular toxicity remains elusive. Our findings suggest that exploiting the protective role of UBE2N by enhancing its expression or activity levels could potentially be a therapeutic strategy. In our study, we have also provided data demonstrating that the protective effects of ramipril are associated with reduced DNA damage downstream of SND1/UBE2N, suggesting that ramipril may be a viable option for mitigating sunitinib-induced vascular damage. Although our data demonstrate that SND1 directly bound to UBE2N and SND1 promoted transcription of UBE2N, the precise mechanism by which SND1 regulates UBE2N requires further investigation. SND1 has been shown to be involved in regulating transcription owing to its role as a transcriptional coactivator (27) and also in posttranscriptional regulation by controlling mRNA stability (86) and mRNA splicing (87), as well as acting as an RNA reader (29). We further found that RNF8 and RNF168 act with UBE2N to promote the recruitment of 53BP1 at sites of DNA lesions. However, the detailed mechanisms underlying the recruitment of 53BP1 by RNF8/RNF168 require further investigation. It should also be noted that while we focused on the DDR pathway in this study, it is plausible that SND1 may regulate other molecular mechanisms that may co-contribute to sunitinib-induced endothelial dysfunction.

We showed that the inhibitory effects of sunitinib on SND1 appear to be independent of the targeting of VEGFR2, PDGFRβ, and c-KIT. Due to sunitinib’s promiscuous targeting of RTKs, identifying the specific kinase responsible for mediating sunitinib’s inhibitory effect on SND1 is complex. Therefore, we attempted to discover a small-molecule compound that could alleviate sunitinib’s toxicity to ECs. Using an in silico drug-repurposing pipeline, we identified ramipril as a candidate that reversed sunitinib-induced EC dysfunction both in vitro and in vivo. Notably, the protective effects of ramipril against sunitinib appeared to be independent of its antihypertensive function, as other classes of antihypertensive drugs were not protective against sunitinib. Instead, our results suggest that the “ACE inhibition” function is more critical, as other commonly used ACE inhibitors exhibit similar protection against sunitinib. The factors that determine why ACE inhibitors with similar chemical structures are able to counteract sunitinib remain to be elucidated. Interestingly, several lines of study appear to support our notion that ACE inhibitors may improve the overall prognosis of cancer patients taking sunitinib. McKay and colleagues reported that among a total of 4,736 metastatic patients with RCC, overall survival (OS) was significantly improved in those on angiotensin system inhibitors (ASIs) compared with users of other antihypertensive agents or receiving no antihypertensive therapy. Importantly, improved OS was observed in ASI users compared with nonusers in individuals receiving anti-VEGF therapy but not temsirolimus or IFN-α (88). Although this study did not specifically focus on anti-VEGF therapy–related toxicities, it is plausible that the improved OS seen in these ASI users is linked to the protective effects of ASI on vascular cells. Coincidentally, a separate study reported that patients receiving anti-VEGF therapy exhibit increased blood pressure, which is reduced by calcium channel blockers and potassium-sparing diuretic agents but not ACE inhibitors (89). These studies agree with our findings that ACE inhibitors may protect patients on anti-VEGF therapy independent of blood pressure control.

There are several limitations in the present study. While the key findings were obtained from hiPSC-ECs, primary ECs, and mouse models, the absence of clinical samples from patients with sunitinib-induced cardiomyopathy limits the translational relevance of our findings. Moreover, patient-specific hiPSCs were not used. Future studies are required to validate the repression of SND1 and UBE2N in patient samples with sunitinib-induced vascular toxicity and also to validate the relevance of the SND1/UBE2N/RNF8/RNF168 axis in predicting individual susceptibility against sunitinib using patient-specific hiPSC-ECs. Although hypertension is prevalent in sunitinib-treated patients, we did not observe this in the mouse model of sunitinib-induced endothelial dysfunction used in this study, likely reflecting the multifactorial causes of hypertension in patients that cannot be modeled in mice. The *C_max_* (peak exposure after a single administration) of sunitinib in clinical use is approximately 0.2 μM; however, a higher dose of 2 μM was used in some experiments, as sunitinib is taken daily for 3 weeks, which leads to accumulation, causing higher exposure at a steady state. Moreover, studies have shown that using in vitro concentrations up to 30 times the in vivo efficacious dose increases the accuracy of toxicity prediction (90, 91). We did perform key experiments using lower concentrations (0.04 and 0.25 μM) of sunitinib.

In summary, our study demonstrates the utility of combining hiPSC-ECs with translatome profiling to identify targets for TKI-induced vascular toxicity. With this approach, we revealed translational regulation of SND1 as a key mediator of sunitinib-induced endothelial dysfunction, which suggests that targeting SND1 translation and the SND1 downstream factors UBE2N, RNF8, and RNF168 is a potential therapeutic strategy for vascular dysfunction. Our study also harnessed the capabilities of an advanced technique, CMap, and identified ramipril as a candidate to prevent sunitinib-induced endothelial dysfunction. This approach may serve as a paradigm for future drug development to treat vascular diseases.

## Methods

### Details are provided in Supplemental Methods

#### Sex as a biological variable.

Our study examined male and female mice, and findings were similar for mice of both sexes.

#### Statistics.

Tests for statistical significance were performed using GraphPad Prism software. Values are presented as mean ± SD. For comparisons between 2 groups, statistical differences were determined by 2-tailed, unpaired Student’s *t* test. For comparison between more than 2 groups, ANOVA with Tukey’s multiple-comparison test was used. *P* values less than 0.05 were considered statistically significant.

#### Study approval.

All animal experiments were approved by the Animal Care and Use Committee of the University of Illinois Chicago (UIC). All experiments were performed in accordance with the relevant UIC guidelines and regulations.

#### Data availability.

Values for all data points in graphs are reported in the Supporting Data Values file. Ribo-Seq data (GSE181278) and RNA-Seq data (GSE219194) are accessible in the Gene Expression Omnibus (GEO) database.

## Author contributions

ZH, ST, and SGO designed and planned the study. ZH, GY, JJ, and SBN performed primary experiments. MA, SK, NT, PVB, and ST performed Ribosome-Seq and data analysis. SZ performed flow cytometry. ZH, GY, JJ, SBN, YK, SZ, JR, SP, SBO, PVB, ST, and SGO analyzed and interpreted the data. ZH, ST, and SGO wrote and revised the manuscript. ZH, GY, JJ, and SBN are co–first authors. Authorship order among co–first authors was determined based on the amount/technical difficulties of the experiments performed. All authors reviewed and approved the manuscript.

## Supplementary Material

Supplemental data

Unedited blot and gel images

Supporting data values

## Version 1 02/03/2025

Electronic publication
