# Supplementary material for: Translational regulation of SND1 governs endothelial homeostasis during stress
Source: J Clin Invest. 2025 Feb 3;135(3):e168730. doi: 10.1172/JCI168730 (PMC11785924; doi:10.1172/JCI168730)
Supplement: Supplemental data [file jci-135-168730-s027.pdf]

Supplementary Materials

Translational Regulation of SND1 Governs Endothelial Homeostasis during Stress

Zhenbo Han, Gege Yan, Jordan Jousma, Sarath Babu Nukala, Mehdi Amiri, Stephen Kiniry, Negar Tabatabaei, Youjeong Kwon, Sen Zhang, Jalees Rehman, Sandra Pinho, Sang-Bing Ong, Pavel V. Baranov, Soroush Tahmasebi, Sang-Ging Ong

Methods

Cell culture. Healthy human iPSC lines were obtained from the Stanford Cardiovascular Institute Biobank. Cells were grown on Matrigel-coated (Corning, 356231) plates using chemically defined E8 medium (Thermo Fischer Scientific). Cells were passaged at a 1:12 ratio every 4 days using accutase solution (Sigma–Aldrich). HEK293T cells were purchased from TakaraBio and cultured in Dulbecco's modified Eagle's medium (DMEM) with high glucose (Thermo Fisher, 11995-065) supplemented with 10% serum and Anti-Anti (Gibco, 15240-062). Immortalized mouse endothelial cells (MECs) were obtained from CELLutions Biosystems (CLU510). The MCECs were grown on 0.1% gelatin-coated plates using EGM-2 Endothelial Cell Growth Media (Lonza, CC-3162). The Primary Aortic Endothelial Cells (HAECs) were obtained from ATCC and grown on 0.1% gelatin-coated plates using EGM-2 Media. The Primary Umbilical Vein Endothelial Cells (HUVECs) were obtained from ATCC and grown on fibronectin-coated plates using EGM-2 Media (Lonza, CC-3162). Human cardiac fibroblasts (HCFs) were purchased from PromoCell and cultured in Fibroblast Growth Medium 3 (PromoCell, C23025).

Differentiation of hiPSC to ECs. The day before differentiation (day 0), iPS cells were dissociated by EDTA and seeded into Matrigel-coated 6 well plate at 1:12 ratio. On the day 1 of differentiation, the cell culture medium was replaced to N2B27 medium consisting of a 1:1 mixture of DMEM:F12 (1:1) (Thermo Fisher Scientific, 11320) and Neurobasal media (Thermo Fisher Scientific, 21103), B27 minus vitamin A (Thermo Fisher, 12587) supplemented with N-2 (Thermo Fisher, 17502), CHIR99021 (8 µM) and BMP4 (25 ng/mL) followed by incubated at 37°C with 5% CO_2 for 3 days. Afterward, media were replaced with StemPro-34 SFM (Thermo Fisher Scientific, 10639011) supplemented with VEGF (100 ng/mL) and forskolin (2 µM). The media were replaced every day for 3 days. On day 7, endothelial cell isolation was performed using Magnetic-activated cell sorting (MACS) system with magnetic microbeads conjugated to CD144 (Miltenyi Biotech, 130-097-857) according to manufacturer's protocol. Sorted hiPSC-EC were maintained in culture using EGM-2 Media (Lonza, CC-3162) in fibronectin-coated well plates at 37°C with 5% CO_2. The medium was changed every 2 days.

Differentiation of hiPSC to CMs. Human induced pluripotent stem cells differentiation to cardiomyocytes (hiPSC-CMs) was achieved using a chemically defined protocol (1). Briefly, hiPSCs at 85% confluency were incubated with differentiation basal medium comprising RPMI 1640 medium (Thermo Fisher, 11875095) and B27 supplement minus insulin (Thermo Fisher, A1895601) supplemented with 6 to 8 µM CHIR99021 for 2 days. Next, the medium was replaced by basal medium and the following day (day 3), the medium was changed to

basal medium supplement with Wnt antagonist, IWR-1 (5 μM). On day 5, the medium switched back to basal medium and maintained for 2 days in this medium. On day 7, the cells were incubated with complete CM medium consisting of RPMI 1640 medium and B27 supplement plus insulin (Thermo Fisher, 17504044) for two additional days. On day 9, the medium was replaced by complete CM medium and the following day, the medium was removed and replaced with glucose starvation medium comprising RPMI 1640 without glucose (Thermo Fisher, 11879020) supplemented with lactate and lipid mixture 1 (Sigma, L0288). On day 12, wells that contain more than 85% beating cells were treated with TrypLE Express (Life Technologies, 12605028) and plated at 1:6 ratio in B27 complete CM medium supplemented 10% KOSR and Y-27632. The next day, hiPSC-CMs were expanded complete CM medium supplemented with 3 μM CHIR99021 and the medium was changed every other day thereafter.

Small molecules. Sunitinib (LC laboratories, S8803), Rapamycin (Selleckchem, S1039), MHY-1485 (Sigma, SML0810), cycloheximide (Sigma, C7698), eFT-508 (Selleckchem, S8275), NSC697923 (Enzo, ENZ-CHM143-0005), Doxycycline hyclate (Thermo, AC446060050), Ramipril (AK Scientific, J90014), Ripasudil (MedChem Express, HY-B0279), Drospirenone (MedChem Express, HY-B0111), Lisinopril (AK Scientific, J54747), Enalapril (AK Scientific, H531), Benazepril (AK Scientific, J94334), Amlodipine (AK Scientific, G697), Atenolol (AK Scientific, E636), CHIR-99021 (Selleckchem, S2924), Y-27632 (Selleckchem, S1049), Forskolin (Selleckchem, S2449), ZM 323881 (Selleckchem, S2896), CP-673451 (AK Scientific, 2336AH), Imatinib (AK Scientific, J10205), BYL-719 (AK Scientific, 2294AH), MK-2206 (AK Scientific, 2181AH).

Virus production. HEK293T cells were co-transfected with lentivector expressing shSND1, shUBE2N, sh4E-BP1, sh4E-BP2, SND1 OE, UBE2N OE, shRNF8, shRNF168, shHLTF, shSHPRH, or a non-targeting control (NTC) (Sigma, SHC216) along with VSV-G envelope plasmid and psPAX2 packaging plasmid using PEI transfection reagent (Polyscience, 24765). Virus containing supernatants were collected at 48 and 72 hours after transfection and concentrated using Lenti-X Concentrator (Takara, 631232). The plasmids overexpressing, human SND1 or UBE2N and control (PLV-Blank) plasmid were purchased from VectorBuilder.

The shRNA plasmids were obtained from Sigma. shRNA sequences for target genes as follows.

shSND1, 5’-TGTGGCTCCCACAGCTAATTT-3’;

sh4E-BP1, 5’- GCGCAATAGCCCAGAAGATAA-3’;

sh4E-BP2, 5’- GCTGTATTTCTGTAGAGCTAA-3’;

shUBE2N, 5’-AGACAAGTTGGGAAGAATATG-3’;

shRNF8, 5’-TGGAGCAACTAGAGAAGACTT-3’;

shRNF168, 5’-GCAGTCAGTTAATAGAAGAAA-3’;

shHLTF, 5’-TGTGGTTGGACTACGCTATTA-3’;

shSHPRH, 5’-CCAGGAGAAATCGCAGTAAAT-3’;

shScramble, 5’-CGTGATCTTCACCGACAAGAT-3’.

Cell viability assays. PrestoBlue reagent (Invitrogen, A13262) were utilized to measure cell viability. In brief, cells were seeded in fibronectin-coated 96-well plate (Greiner Bio-One, 655098 or 655090) at 3000 cells per well and incubate in incubator for 24h. Cells were treated with different drugs or transfected with viruses. Afterward, 10 µL of PrestoBlue reagent were added to each well. Then cells were incubated for 2 h at 37°C. And the fluorescence excitation wavelength of 560 nm and an emission of 590 nm were measured by FlexStation 3 microplate reader (Molecular Devices).

Tube formation assay. The tube formation assay was performed by using µ-Slide Angiogenesis dish (Ibidi, 81506) according to manufacturer’s instructions. 10 µL of Matrigel (Corning, 354277) was added to each dish and incubated at 37°C for one hour. During the incubation period, the hiPSC-ECs, HUVECs, and HAECs were seeded into each dish at density of 10000 cells/well. The cells were then incubated at 37°C for 16 hours. Images were then recorded by Echo Revolution microscope and the data were analyzed using the ImageJ software (National Institutes of Health, https://imagej.nih.gov/ij/).

Wound healing assay. HiPSC-ECs, HUVECs, and HAECs were seeded onto 24 well plates at a density of 100000 cells/well and incubate for 24h. Scratches were made using a 200 µL sterile pipette tip. Images were recorded after 0 and 16 hours after scratch. The images were analyzed by MRI wound healing tool plugin from imageJ software (National Institutes of Health, http://rsb.info.nih.gov/ij/).

RNA Sequencing (RNA-Seq). Total RNAs was extracted by Direct-zol RNA Miniprep kit (Zymo, R2052) with DNase I treatment. mRNA was purified by using poly-T oligo-attached magnetic beads. The NEBNext Ultra RNA Library Prep Kit for Illumina (NEB, USA) was used for RNA-Seq library construction. In brief, 1 µg of RNA was fragmented in 94°C for 5 min. Then the first and second strand cDNA was synthesized by provided reagents. To preferentially select cDNA fragments between 150 and 200 bp, the AMPure XP system (Beckman Coulter,

Beverly, USA) was used to purify the library fragments. The size-selected, adaptor-ligated cDNA was treated with the USER Enzyme (NEB, USA) at 37°C for 15 min and 95°C for 5 min before PCR. PCR was then carried out with Phusion High-Fidelity DNA polymerase, Index (X) Primer and Universal PCR primers. Finally, the Agilent Bioanalyzer 2100 system was used to purify the PCR products (AMPure XP system), and assess the library quality. Libraries were sequenced with Illumina HiSeq 2500. BioJupies web server (https://maayanlab.cloud/biojupies/), a cloud-based analysis suite, was used for mapping and QC for RNA-seq raw data (2). The ontological analysis was performed using EnrichR web server (https://maayanlab.cloud/Enrichr/) (3). The Heat maps were generated by Morpheus (Broad institute). Sequencing data has been uploaded (accession number: GSE219194) and will be made publicly available upon acceptance of manuscript. Temporary reviewer token: qnqrkaiwzdsbjit.

Ribosome profiling (Ribo-Seq). The ribosome profiling was performed as described before (4), with minor modifications. Briefly, hiPSC-ECs (two biological replicates) were treated with sunitinib or control for 24 hrs. Next, cells were incubated with cycloheximide (CHX) at a final concentration of 100 µg /ml for 5 min at 37°C incubator and then washed twice with ice-cold PBS containing 100 µg/mL CHX. Following scraping and centrifugation, cell pellets were resuspended in lysis buffer (20 mM Tris [pH 7.4], 5 mM MgCl2, 100 µg/ml of cycloheximide, 150 mM NaCl, 1 mM DTT, 500 U/mL RNase inhibitor, 25 U/mL Turbo DNase I and Protease Inhibitor). 1% v/v Triton X-100 was added to cell lysis and incubated on ice for 10 min. The lysate was centrifuged at 16000 g for 10 min at 4°C, and a portion of the supernatant was reserved for the preparation of an RNA-Seq library. The rest of the lysate was digested with RNase I (6U RNase I (Epicenter) per A260 Unit for 45 min at RT). RNase I digestion was terminated by incubation with SUPERaseIn for 5 min. Samples were then loaded on a sucrose cushion (1 M sucrose and 20 U/ml SUPERaseIn) and centrifuged at 70,000 rpm for 3 h. RNA fragments were extracted with TRIzol reagent. Purified RNA samples were resolved on 15% TBU-Urea gel, and fragments in range of 17-34 nt were excised, eluted, and precipitated with isopropanol. We performed rRNA depletion (using NEBNext RNA Sample Depletion kit (E6350S)) and fragmentation (using NEBNext Magnesium RNA Fragmentation kit (E6150)) on total RNA sample before both footprint fragments and total RNA fragments were subjected to dephosphorylation (using T4 Polynucleotids Kinase (T4 PNK, NEB)) and linker ligation. The purified ligated RNA samples were reverse-transcribed, and cDNA was then circularized using CircLigase I followed by another round of rRNA depletion using biotin-labelled antisense probes and Dynabeads MyOne Streptavidin C1. The final PCR

reaction was done using Phusion polymerase, and the PCR product was purified using a DNA clean & concentrator column according to the manufacturer instruction. Purified PCR products were separated on an 8% TBE gel. The bands corresponding to ~160 bp were excised and extracted using the DNA extraction buffer described above. RNA-Seq libraries was prepared at UC Davis. The sequencing was carried out at the DNA Technologies and Expression Analysis Core at the UC Davis Genome Center, supported by NIH Shared Instrumentation Grant 1S10OD010786-01.

Analysis of Ribosome-Profiling data. Sequenced libraries were processed by removing the adapter using Cutadapt 3.4 toolkit (5). Trimmed reads were further processed by discarding low-quality reads and reads shorter than 25 nt. The reads were aligned to rRNA and tRNA using Bowtie 1.3.0 (6). PCR duplicates were removed using UMI-Tools 1.0.1 (7) and the remaining reads were then aligned to human Gencode version 25 comprehensive transcriptome. The final BAM file was converted to an SQLite file compatible with Trips-Viz (https://trips.ucc.ie/), a platform for the Ribo-Seq data analysis (8). The quality control analysis (Metagene, and correlation analysis was done using built-in functions of Trips-Viz. MTRNR2L1 (N1f) pseudogene was excluded from Ribo-Seq 5'UTR/CDS/3'UTR distribution analysis. Differential expression analysis was carried out using Z-score analysis was carried out as previously described (8). Sequencing data has been uploaded (accession number: GSE181278) and will be made publicly available upon acceptance of manuscript. Temporary reviewer token: wtkjeqsipncddez.

Western Blotting. The cell lysates were extracted by using RIPA buffer (Sigma, R0278) supplemented with protease and phosphatase inhibitor (Thermo Fisher, 78440). Pierce Rapid Gold BCA Protein Assay Kit (Thermo Scientific, A53226) was used for Protein quantification. Proteins were separated via Bis-Tris gels and transferred to PVDF membranes (Thermo Scientific, 88520) using wet-based transfer system (Bio-Rad). Membranes were incubated for 1 hour at room temperature in blocking buffer (Thermo Scientific, 37536) and with the indicated primary antibodies overnight at 4°C followed by incubation for 2 hours with secondary antibodies (Cell Signaling Technology, 7074S and 7076S). Signals were detected using iBright imaging system (Thermo Fisher Scientific). The results were analyzed using iBright Analysis Software (Thermo Fisher Scientific).

Antibodies. Antibodies used were as follows: SND1 (Invitrogen, PA5-89222, WB/IHC), SND1 (Invitrogen, PA5-40124, Co-IP), SND1 (Proteintech, 10760-1-AP, Flow), p4E-BP1 (CST, 9451), 4E-BP1 (CST, 9644), UBE2N (Invitrogen, 37-1100), Ubiquitin (Invitrogen, 13-1600), K63 (CST, 5621), Puromycin (Millipore,

MABE343), p-mTOR (CST, 5536S), mTOR (CST, 2983S), p-AKT (Invitrogen, 44-602G), AKT (CST, 4691S), p-S6 (CST, 4858S), S6 (CST, 2217S), pEIF4E (Invitrogen, 44-528G), p-eNOS (Invitrogen, MA5-14957), eNOS (Invitrogen, PA1-037), GAPDH (Proteintech, 60004-I-Ig), α-Actinin (Sigma, A7811), p-VEGFR2 (Invitrogen, 441047G), VEGFR2 (Invitrogen, MA5-15157), 53BP1 (Invitrogen, PA1-16565), Phalloidin (Thermo, F432), VE-Cadherin (R&D, AF938), CD31 (R&D, AF806), γ-H2A.X (Millipore, 05-636), RNF8 (Invitrogen, PA5-20961), RNF168 (Invitrogen, PA5-31119), Ki-67 (Invitrogen, MA5-14520), F4/80 (Invitrogen, 25-4801-82), CD19 (Invitrogen, RM7705), CD45 (Invitrogen, 25-0451-81), CD31 (Invitrogen, 120311-81, Flow), MHC II (Invitrogen, 12-5321-81), CD45 (Invitrogen, 17-0451-82), CD11b (Invitrogen, A15390), Ly-6C (Invitrogen, 45-5932-80), CD3e (Invitrogen, 45-0031-80), CD4 (Invitrogen, A15384), Donkey anti-Rabbit IgG Alexa fluor 594 (Invitrogen, A21207), Donkey anti-Rabbit IgG Alexa fluor 488 (Invitrogen, A21206), Donkey anti-Mouse IgG Alexa fluor 594 (Invitrogen, A21203), Donkey anti-Mouse IgG Alexa fluor 488 (Invitrogen, A21202), Anti-Rabbit-HRP (CST, 7074S), and Anti-Mouse-HRP (CST, 7076S).

Co-Immunoprecipitation (Co-IP). Co-Immunoprecipitation was performed using the pierce Co-IP Kit (Thermo Fisher Scientific, 26149) according to the manufacturer's instruction. In brief, hiPSC-ECs were grown in 10-cm dish to reach 90% confluence, the cells were harvest and lysed in ice cold IP lysis buffer (0.025M Tris, 0.15M NaCl, 0.001M EDTA, 5% glycerol, 1% NP-40; PH 7.4) containing phosphatase inhibitors (Thermo Fisher Scientific, 78440). 10% of cell lysis was saved as input control. 20 μg of anti-Strep (Abcam), anti-UBE2N (Invitrogen, 37-1100), and anti-IgG (Invitrogen) were pre-immobilized with coupling resin. Cell lysis was mixed with antibody-coupled resin overnight at 4°C with continuous rocking. The protein was eluted in 50 μL of elution buffer for western blot assay. Signals were detected using iBright imaging system (Thermo Fisher Scientific) and results were analyzed using iBright Analysis Software (Thermo Fisher Scientific).

Surface Sensing of Translation (SUnSET) assay. To examine protein synthesis in hiPSC-ECs, cells were treated with sunitinib for 24 hours. Cells were then incubated with 1 μM puromycin for 2 hours. The hiPSC-ECs were incubated with global protein synthesis inhibitor cycloheximide (Sigma, C7698) at a final concentration of 100 μg/ml along with puromycin. Then protein was extracted for immunoblotting with anti-puromycin antibody (Millipore, MABE343).

Glucose-6-Phosphate Dehydrogenase release assay. Glucose-6-Phosphate Dehydrogenase (G6PD) is a cytosolic enzyme that leaks out when plasma membrane integrity of cells is compromised. G6PD release assay kit (V23111; Thermo Fisher Scientific) was used for detecting G6PD according to the manufacturer's instructions.

Immunofluorescence. Cells seeded on fibronectin-coated microscope coverslips (Thermo Fisher Scientific) were fixed with 4% paraformaldehyde (EMS, 15710) for 15 min at room temperature followed by permeabilized with 0.5% triton X-100 (Sigma, 93443) for 10 min at room temperature. Cells were then blocked with 5% donkey serum for 1 h. Primary antibody (diluted in blocking buffer) incubation was performed overnight at 4 °C. After 3 times washing with PBS, the cells were incubated with secondary antibodies (diluted in blocking buffer) and DAPI for 1 hour at room temperature. Cells were mounted in PermaFluor™ mounting medium (Epredia, TA-030-FM). Immunostaining images were captured using Zeiss LSM 880 confocal microscopy. Images were analyzed using ZEN 3.4 Microscopy Software (Zeiss).

RNA extraction and real-time polymerase chain reaction (PCR). Total RNA was isolated from cells by using Direct-Zol RNA MiniPrep (Zymo Research, R2052) and treated with DNase using PureLink DNase Set (Thermo Fisher Scientific, 12185010) according to manufacturer's instructions. The reverse transcription was performed by High-Capacity cDNA Reverse Transcription Kit (Invitrogen, 4374966). Quantitative real-time PCR was performed with PowerUp SYBR Green Master Mix (Applied Biosystems, A25741) on a QuantStudio Flex Real-Time PCR System (Applied Biosystems). Primers that were used are as followed: *hSND1* (forward: GGTGGACTACATTAGACCAGCC; reverse: AGACCTTTGCTGACAAGAGCCTC), *hUBE2O* (forward: AACATCCGCTCCAACGACCTCT; reverse: CCAGACTGTACCACACCGTAGA), *hFBXO25* (forward: ATTCTCGCCTGGCAACAACAGC; reverse: GATGTCCCATCCGTCTGAGAAC), *hERCC8* (forward: GCAGTTTCCTGGTCTCCACGTT; reverse: CAAACATCCTGATGCTCTTCTCAC), *hCDC73* (forward: GAGAGAGTATGGAGGACACGAAC; reverse: ATTTGGGGCAGGTCGCTGTTCA), *hRMND5A* (forward: CATCCACAGCAGTGTTTCTCGG; reverse: CGAAAGAAGTGCTCCACCATCAC), *hFBXO9* (forward: ATAGAGCCTGGCACCAAGTGGA; reverse: GGAACAATGGACTGAGGCTCTTC), *hKBTBD7* (forward: AGTCTCTGGTGCCAGTGCCAAA; reverse: CCATCTCCTTGGCACACATACC), *hUBE2N* (forward: TGATGTAGCGGAGCAGTGGAAG; reverse: GGAGGAAGTCTTGGCAGAACAG), *hGAPDH* (forward: TCAAGGCTGAGAACGGGAAG; reverse: GGACTCCACGACGTACTCAG).

TCGA analysis of SND1 within different tumors. We used GEPIA2 (Gene expression profiling interactive analysis, version 2) web server (http://gepia2.cancer-pku.cn/#index) to analyses the correlation between SND1 with different tumors. To observe the expression difference of SND1 between normal tissues and tumor tissues for the different type of tumors. In box plot module, we input SND1 as selected gene, and setup the parameters with |Log2FC|=1 and p-value Cutoff=0.01, then output the data. The expression of SND1 in different stages (stage I, stage II, stage III, and stage IV) of tumors were analyzed by using stage plot module in GEPIA2. To obtain the overall survival data of SND1 in different tumors, in survival analysis module, we input SND1 as selected gene, and setup the parameters with Cutoff-High(%)=50 and Cutoff-Low(%)=50.

Live/Dead cell assay. HCS LIVE/DEAD Green kit (Invitrogen, H10290) was used for live/dead cell staining according to the manufacturer's instructions. Images were captured using ECHO revolve (ECHO) microscopy.

Identification of Ace-Inhibitor Ramipril. A systems pharmacology approach was performed by using a subset of the most differentially regulated genes identified by RNA-Seq (150 upregulated, 150 downregulated) as determined by LogFC, which also contained p-values <0.05. These genes were uploaded to the Connectivity Map and compared to over one million transcriptomic signatures, which are contained within the L1000 Touchstone database (9, 10). Query results were then exported and filtered to include signatures derived from only chemical compounds and ranked based on raw connectivity score and transcriptional activity score which is a measure of signature strength and replicate correlation assigned for a given connection.

Experimental animals. C57B/L6 mice (#000664) and NU/J immune deficient nude (#002019) were purchased from Jackson Laboratory. Mice were housed under constant temperature and humidity and fed with standard diet. All experimental mice were selected with similar age (8-10-week-old) and body weights.

Immunohistochemical staining of the aorta tissue. C57BL/6 mice (8-10-week-old) were orally administrated with DMSO or sunitinib at a dose of 40 mg/kg daily for 3 weeks. Aorta were collected and fixed in formalin, embedded in paraffin. Tissues were sectioned for 5 μm and mounted on slides. Sections were deparaffinized with xylenes before dehydrated with ethanol. Antigen retrieval was performed with citrate buffer. The sections were then blocked with 10% goat serum followed by incubating with anti-SND1 antibody (Invitrogen, 82020) overnight at 4˚C. Sections were incubated with secondary antibody. Then slides were visualized by 3,3′-Diaminobenzidine (DAB) with DAB substrate kit (Life technology, 34002). Slides were stained with hematoxylin

and eosin (H&E). Images were taken with a Leica DMi8 microscope (Leica microsystems) and analyzed using the ImageJ software (National Institutes of Health, http://rsb.info.nih.gov/ij/).

Isolation of mice cardiac endothelial cells (MCECs). The MCECs were isolated from C57BL/6 mice heart as previously described (11-13) with some modifications. Briefly, heart tissues were minced into ~1 mm^2 pieces and digested in collagenase II (2mg/mL dissolved in DMEM) for 50 min at 37°C. Cell suspensions were filtered through 40 μm filter and then neutralized in neutralization media (10% FBS in DMEM). The suspensions were centrifuged at 1200 rpm for 8 min at 4°C. The cell pellet was washed and incubated with CD31-coated dynabeads for 20 min at room temperature. Beads were washed and bead-bound cells were resuspended in appropriate volume of EGM2 medium or RIPA buffer for further analysis.

Immunostaining of heart tissue. Mice were humanely euthanized, and the hearts were harvested. Tissue samples were fixed using 4% paraformaldehyde and subsequently embedded in OCT compound. The tissues were sectioned into slices with a thickness of 10 micrometers. The slides were washed three times in PBS. Tissue sections were blocked with 5% BSA in PBS (containing 0.2% Triton X-100) for 1h at room temperature and then incubated with primary antibodies at 4°C overnight. The tissue sections were then washed with PBS three times and incubated with Alexa-conjugated secondary antibodies (Invitrogen) for 1h at room temperature. Sections were washed in PBS for 15 min and mounted with mounting medium (Thermo Fisher Scientific). Images were captured using Zeiss LSM 880 confocal microscopy. Images were analyzed using ZEN 3.4 Microscopy Software (Zeiss).

Adenovirus or Adeno-associated virus production for SND1 knockdown in vivo. Adeno-associated virus serotype 9 (AAV9) carrying intercellular adhesion molecule 2 (ICAM-2) promoter driving SND1-shRNA (target sequences: CCTCAAGTACACCATTGAGAA) or scramble shRNA were constructed and packaged by Gene Universal Inc. We introduced the viruses into the C57BL/6 mice (8 weeks old, both gender) via tail vein injection at a dose of 10^{11} VG/mice. We isolated mouse cardiac endothelial cells and verified the knockdown efficiency of SND1 in endothelial cells by immunoblotting.

In vivo matrigel plug assay. Mouse endothelial cells were transduced with either i) control vector, ii) shSND1, iii) SND1 overexpression lentiviral vectors. Two days after transduction, cells were labeled with Vybrant CM-DiI Cell-Labeling Solution (Invitrogen, v22888) for 20 min. Before injection, growth factor reduced matrigel (Corning, 356231) was premixed with VEGF (vascular endothelial growth factor, 50 ng/mL). Cells were then

centrifuged and resuspended by 500 μL of matrigel followed by inject into flank regions of C57BL/6 mice (8-10-week-old). Seven days after injection, mice were euthanized and matrigel plugs were removed. After fixed with formalin overnight at 4˚C, plugs were dehydrated and then embedded with paraffin, and sectioned (10 μm) by rotary microtome (Thermo). Sections were mounted onto histological slides and immunostained with CD31 antibody. Images were taken with a Leica DMi8 microscope (Leica microsystems).

Hemoglobin content assay. Hemoglobin content of matrigel plugs was determined by hemoglobin assay kit (Chondrex, 6024) according to manufacturer's instructions. In brief, one vial of standard was reconstituted with 1 mL of distilled water to make 2 mg/mL standard solution. Then the standard was diluted in sequence to make 0.5, 0.25, 0.125, 0.063 and 0.031 mg/mL solutions. The matrigel plug tissues were dissolved in distilled water and homogenized using TissueRuptor II (QIAGEN, 9002755). The samples were centrifuged at 10000 RPM for 6 min at 4˚C and the supernatant was collected for analyze. Then 50 μL of standard or sample solutions were added to the wells in duplicate before adding 200 μL of reaction solution. Finally, the plate was read at 400 nm absorbance using FlexStation 3 Multimode Plate Reader (Molecular Devices, LLC).

Flow cytometry. Mice hearts were harvested and perfused with cold PBS and minced into small pieces. The tissue was digested in collagenase II (2mg/mL dissolved in DMEM) for 50 min at 37°C. Cell suspensions were filtered through 40 μm filter to remove cardiomyocytes and then neutralized in neutralization media (10% FBS in DMEM). The suspensions were centrifuged at 300 g for 10 min at 4°C. The cells were stained with indicated antibodies. Data were acquired on a BD FACSCelesta flow cytometer (BD Biosciences) with FACSDIVA software (BD Biosciences). Data were analyzed using Flowjo software (FlowJo, LLC).

786-O-Fluc cell culture and orthotopic xenografts. Luciferase labeled ccRCC 786-O cell line (786-O-Fluc) (a kind gift by Dr. William Kaelin, Dana-Farber Cancer Institute, Boston, MA, USA) were cultured in DMEM containing 10% FBS in 5% CO_2 at 37 °C. The immune deficient nude mice in both gender (3 Male and 3 Female each experimental group) were used for orthotopic xenograft. Briefly, after the right kidney was exposed, 1×10^6 of cell suspensions (20 μL in PBS) were injected into subrenal capsule using a 27-gauge needle. Mice were monitored daily for signs of distress. The tumors were monitored weekly by BLI imaging. The tumors were allowed to reach a photon emission of approximately ~10^7 photons/s (2 to 3 weeks post-xenograft) prior to initiating daily oral administration of either sunitinib alone (40 mg/kg per day), ramipril alone (10 mg/kg/day), sunitinib plus ramipril, or the vehicle, maintained for a period of 3 weeks. At the end of the experiment, mice were

euthanized, tumors were collected, and the tumor volume was calculated using the formula: length × width^2 × 0.52. Tumors were then fixed in 10% paraformaldehyde before embedded in paraffin. Tumor tissues were then sectioned into five micron-thick and immunohistochemistry was performed using anti-Ki-67 antibody (Invitrogen, MA5-14520).

Measurement of CFR. The CFR was measured using the Vevo2100 imaging system (Visual Sonics Inc, Canada). Mice were placed into an induction chamber, and anesthesia was induced using 3% isoflurane. Hind paw reflexes were briefly assessed to evaluate the level of consciousness. Unconscious mice were then secured to a warming platform to ensure a constant body temperature of 37°C. Isoflurane was then reduced to 1%. The MS550D transducer with a center frequency of 40 MHz was then used to obtain a bright field view of the parasternal long-axis. The septal coronary artery (SCA) was then visualized using the Color-Doppler function. Baseline recordings of coronary flow rates were then obtained, whereafter isoflurane concentrations were increased to 2.5% to induce a hyperemic state. Peak blood flow velocities were then measured and recorded in both the baseline and hyperemic states. The CFR was calculated as the ratio of peak velocity during hyperemia (2.5%) relative to the baseline (1%) state. Measurements consisted of at least three consecutive peaks recorded from consistent waveforms.

References

1. Buikema JW, et al. Wnt Activation and Reduced Cell-Cell Contact Synergistically Induce Massive Expansion of Functional Human iPSC-Derived Cardiomyocytes. *Cell Stem Cell.* 2020;27(1):50-63 e5.
2. Torre D, et al. BioJupies: Automated Generation of Interactive Notebooks for RNA-Seq Data Analysis in the Cloud. *Cell Syst.* 2018;7(5):556-61 e3.
3. Kuleshov MV, et al. Enrichr: a comprehensive gene set enrichment analysis web server 2016 update. *Nucleic Acids Res.* 2016;44(W1):W90-7.
4. McGlincy NJ, Ingolia NT. Transcriptome-wide measurement of translation by ribosome profiling. *Methods.* 2017;126:112-29.
5. Kechin A, et al. cutPrimers: A New Tool for Accurate Cutting of Primers from Reads of Targeted Next Generation Sequencing. *J Comput Biol.* 2017;24(11):1138-43.
6. Langmead B, et al. Ultrafast and memory-efficient alignment of short DNA sequences to the human genome. *Genome Biol.* 2009;10(3):R25.
7. Smith T, et al. UMI-tools: modeling sequencing errors in Unique Molecular Identifiers to improve quantification accuracy. *Genome Res.* 2017;27(3):491-9.

8. Kiniry SJ, et al. Trips-Viz: an environment for the analysis of public and user-generated ribosome profiling data. *Nucleic Acids Res.* 2021;49(W1):W662-W70.
9. Lamb J, et al. The Connectivity Map: Using Gene-Expression Signatures to Connect Small Molecules, Genes, and Disease. *Science.* 2006;313(5795):1929-35.
10. Subramanian A, et al. A Next Generation Connectivity Map: L1000 Platform and the First 1,000,000 Profiles. *Cell.* 2017;171(6):1437-52.e17.
11. Sawada N, et al. Regulation of endothelial nitric oxide synthase and postnatal angiogenesis by Rac1. *Circ Res.* 2008;103(4):360-8.
12. Yucel N, et al. Cardiac endothelial cells maintain open chromatin and expression of cardiomyocyte myofibrillar genes. *Elife.* 2020;9.
13. Lim YC, et al. Heterogeneity of endothelial cells from different organ sites in T-cell subset recruitment. *Am J Pathol.* 2003;162(5):1591-601.

Supplemental Figures

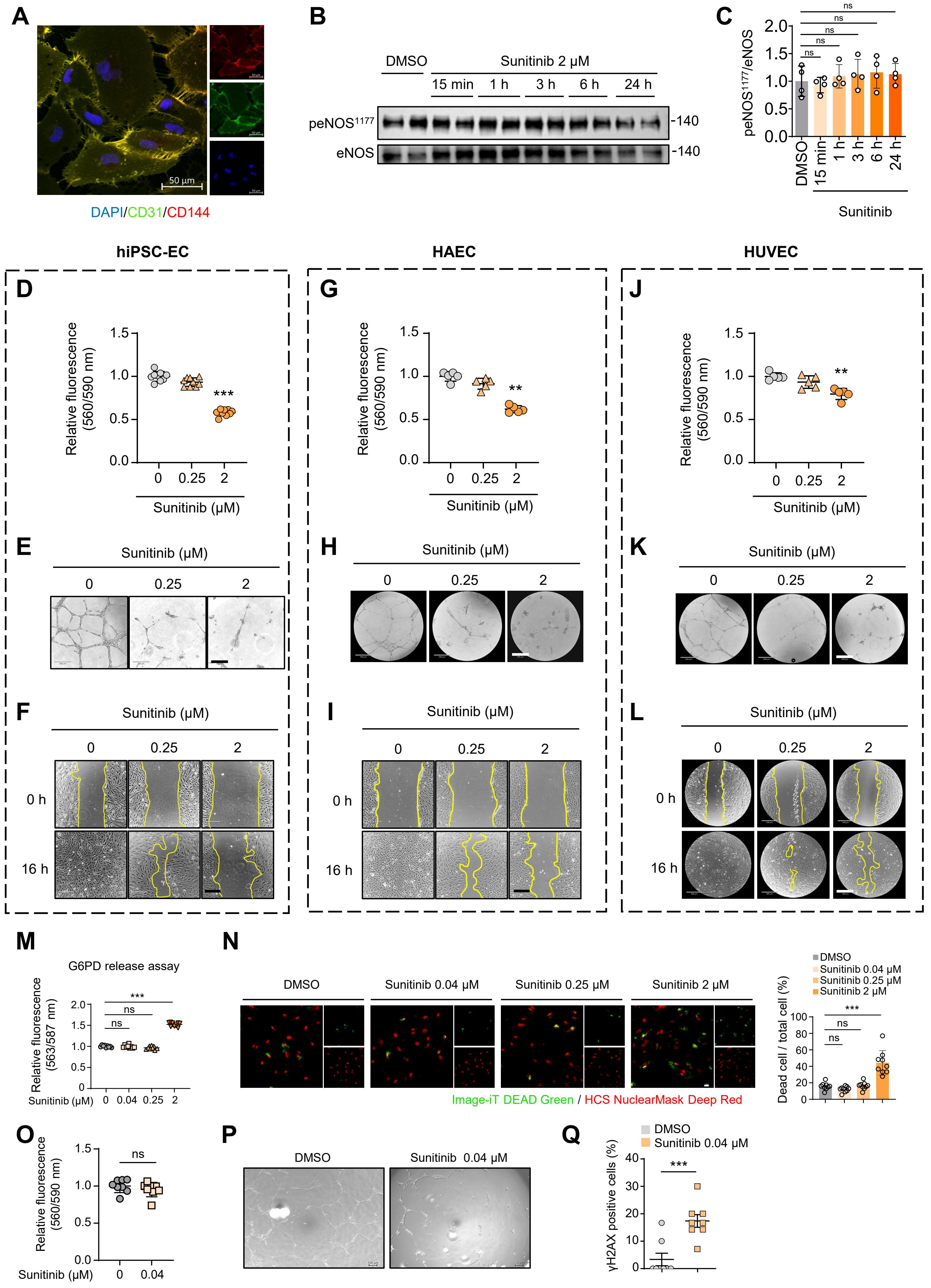

Supplemental Figure 1

Supplemental Figure 1. Detrimental effects of sunitinib in ECs. (A) Differentiated hiPSC-ECs stained positive for CD31 and CD144. Scale bar, 50 μm. **(B** and **C)** hiPSC-ECs were treated with sunitinib (2 μM). Immunoblot analysis indicated sunitinib did not affect p-eNOS/eNOS ratio. One-way ANOVA. N=4 technical replicates. NS; not significant. **(D-F)** The effects of sunitinib (0.25 and 2 μM) on hiPSC-EC viability and function was determined by PrestoBlue viability assay (n=9 replicates from the differentiation of three individual hiPSC lines), tube formation assay and scratch assay. One-way ANOVA. Data are presented as mean ± SD. ***P<0.001. Scale bar, 220 μm. **(G-I)** The effects of sunitinib (0.25 and 2 μM) on HAEC viability and function was determined by PrestoBlue viability assay (n=5 technical replicates), tube formation assay (Scale bar, 340 μm) and scratch assay (Scale bar, 220 μm). One-way ANOVA. Data are represented as mean ± SD. **P<0.01. **(J-L)** The effects of sunitinib (0.25 and 2 μM) on HUVEC viability and function was determined by PrestoBlue viability assay (n=5 technical replicates), tube formation assay (Scale bar, 340 μm) and scratch assay (Scale bar, 860 μm). One-way ANOVA. Data are presented as mean ± SD. **P<0.01. **(M)** G6PD release assay of hiPSC-ECs indicated that only high concentration (2 μM) of sunitinib could causes cytotoxicity. One-way ANOVA. Data are presented as mean ± SD. ***P<0.001. NS; not significant. N=9 replicates from the differentiation of three individual hiPSC lines. **(N)** Representative immunostaining of hiPSC-ECs stained with Image-iT DEAD Green stain (green) and HCS NuclearMask™ Deep Red (red) after treatment with DMSO or sunitinib (0.04, 0.25, and 2 μM) for 48 h. One-way ANOVA. Data are presented as mean ± SD. ***P<0.001. NS; not significant. N=9 replicates from the differentiation of three individual hiPSC lines. Scale bar, 20 μm. **(O)** hiPSC-ECs were treated with 0.04 μM of sunitinib for 72 hrs. Cell viability was determined using the PrestoBlue cell viability reagent. Two-tailed Student's t-test. Data are presented as mean ± SD. ns; not significant. N=8 replicates from the differentiation of two individual hiPSC lines. **(P)** hiPSC-ECs were treated with 0.04 μM of sunitinib for 72 hrs. Endothelial cell function was determined by tube formation assay. Scale bar, 100 μm. **(Q)** The quantification of γ-H2AX/CD31 staining in hiPSC-ECs after sunitinib treatment (0.04 μM) for 72 hrs. Two-tailed Student's t-test. Data are presented as mean ± SD. ***P<0.001. N=8 replicates from the differentiation of two individual hiPSC lines.

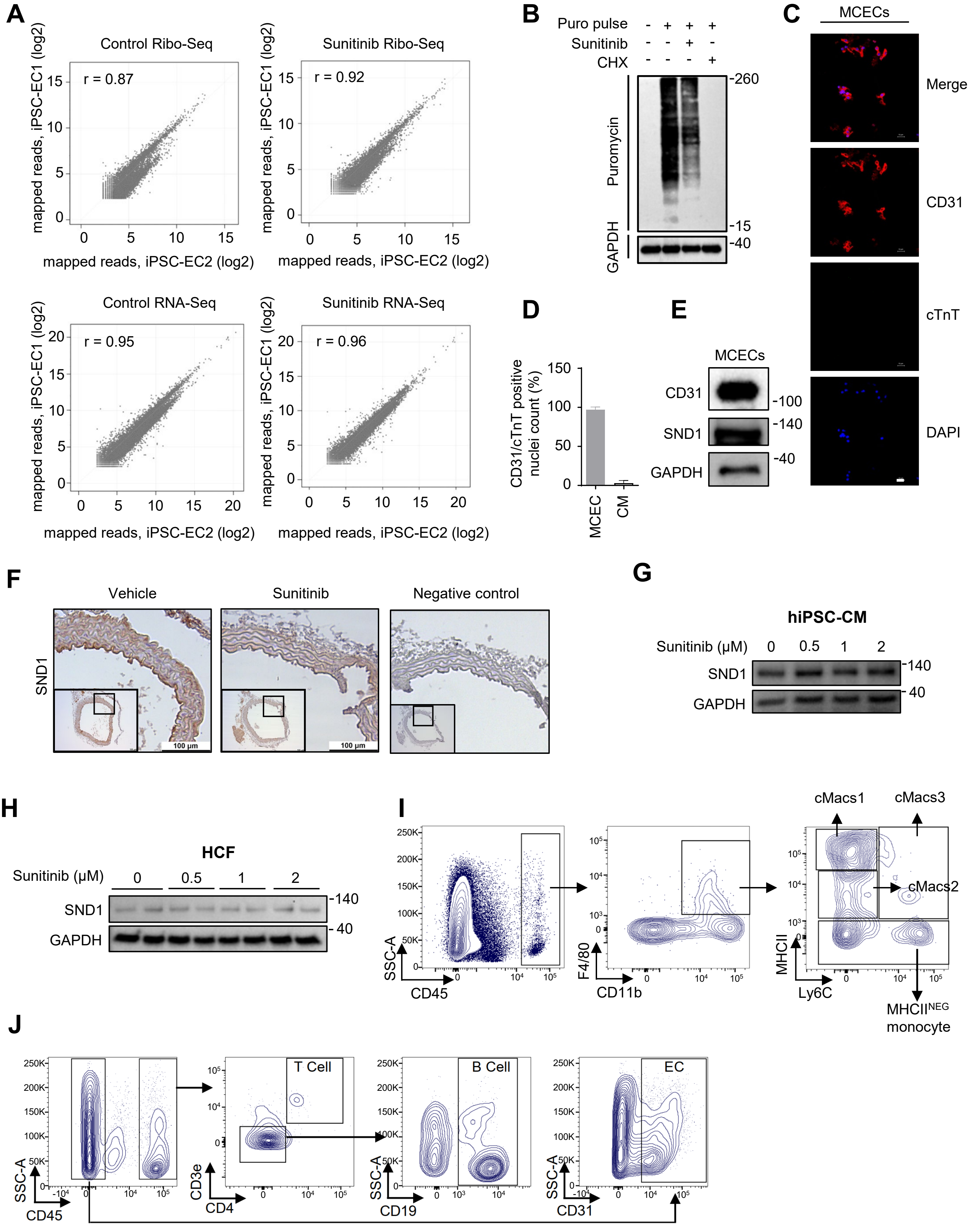

Supplemental Figure 2

Supplemental Figure 2. Sunitinib inhibits SND1 protein level in endothelial cells. (A) Correlation plots of ribosome profiling experiments performed on two independent hiPSC-EC lines treated with DMSO (control) or sunitinib. **(B)** hiPSC-ECs were treated with 10 μM puromycin after sunitinib or DMSO treatment. Puromycin incorporates into newly protein synthesis. Immunoblot analysis with an antibody against puromycin indicated sunitinib treatment inhibits *de novo* protein synthesis. Cycloheximide (CHX; an inhibitor of translation elongation) was used as a positive control **(C** and **D)** Immunostaining and quantification of CD31 and cTnT positive cells in isolated mouse cardiac endothelial cells. N=4. **(E)** Immunoblot analysis of CD31 and SND1 level in isolated mice cardiac endothelial cells (MCECs). **(F)** Immunohistochemical staining for SND1 revealed sunitinib treatment caused a noticeable downregulation of SND1 expression in the mice aortic endothelium. Negative control: no primary antibody incubation. Scale bar, 100 μm. **(G)** hiPSC-CMs (cardiomyocytes) were treated with different concentrations of sunitinib for 48 hrs. Immunoblot analysis indicated sunitinib treatment did not affect SND1 level in hiPSC-CMs. **(H)** Human cardiac fibroblasts (HCFs) were treated with different concentrations of sunitinib for 48 hrs. Immunoblot analysis indicated sunitinib treatment did not affect SND1 level. **(I)** Gating strategy used to identify cardiac macrophage subsets and monocyte by flow cytometry. **(J)** Gating strategy used to identify $CD4^+$ T cells, $CD19^+$ B cells and $CD31^+$ endothelial cells by flow cytometry.

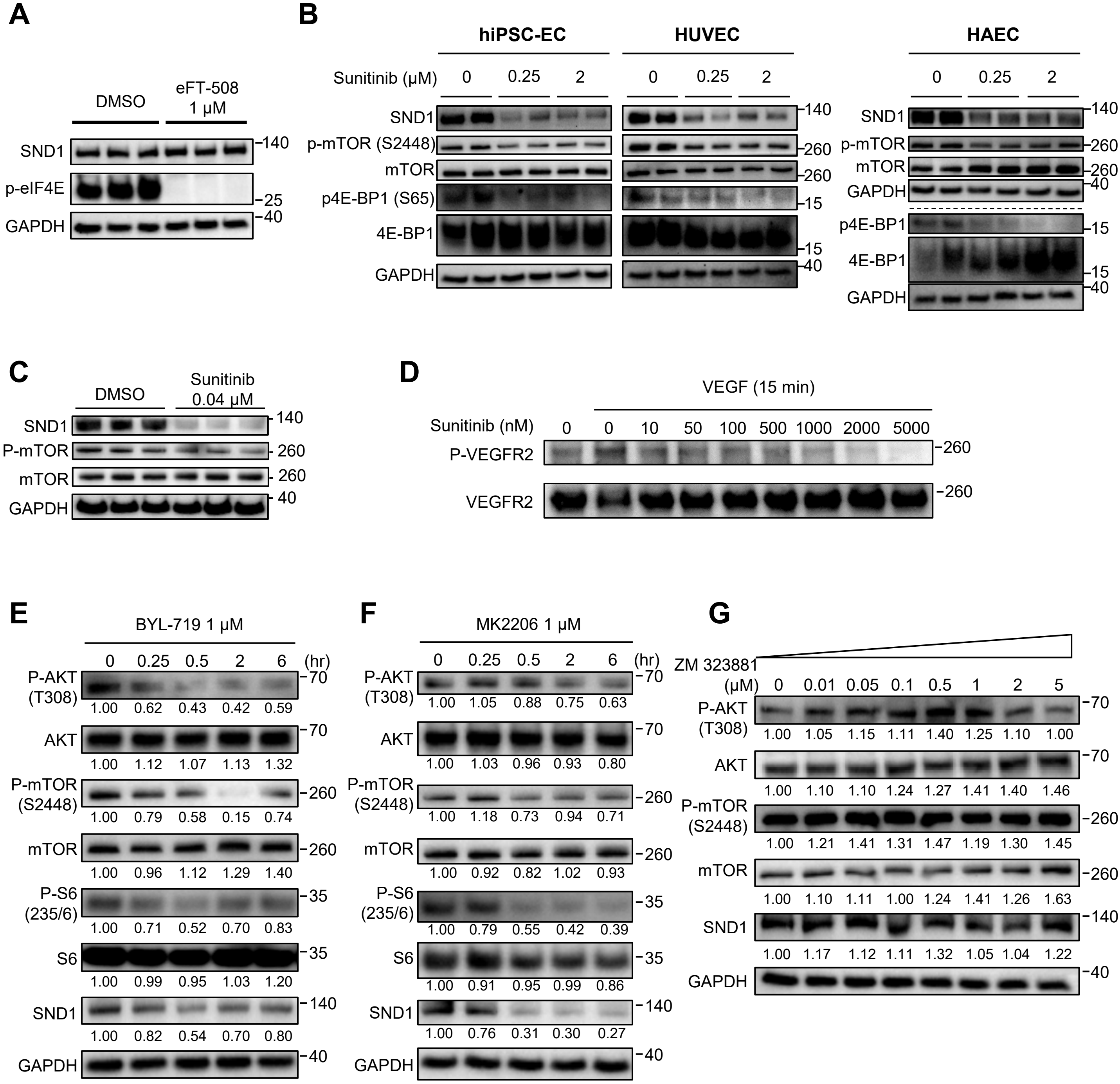

Supplemental Figure 3

Supplemental Figure 3. Sunitinib translationally inhibits SND1 via mTOR/4E-BP1 pathway. (A) hiPSC-ECs were treated with eFT-508 (1 μM), a potent inhibitor of MNK1 and MNK2. Immunoblot analysis indicated eFT-508 blocked phosphorylation of eIF4E (a known target of MNK1/2) but did not affect SND1 level. **(B)** Representative immunoblot analysis of mTOR and its downstream target, 4E-BP1, in hiPSC-ECs, HUVECs, and HAECs treated with sunitinib (0.25 and 2 μM) revealed reduced SND1, phospho-mTOR and phospho-4E-BP1. **(C)** Representative immunoblot analysis of SND1 and mTOR in hiPSC-ECs treated with sunitinib (0.04 μM) revealed reduced SND1 and phospho-mTOR. **(D)** After 16 h of serum starvation, hiPSC-ECs were exposed to 10 nM VEGF165 with or without indicated concentrations of sunitinib. After 15 min of incubation, cells were lysed, and immunoblot analysis was performed to detect p-VEGFR2 or VEGFR2 expression. **(E)** Immunoblot analysis indicated that BYL-719 (1 μM) treatment inhibits SND1 expression and suppresses the phosphorylation of AKT, mTOR, S6 in hiPSC-ECs. **(F)** Immunoblot analysis indicated that MK2206 (1 μM) treatment inhibits SND1 expression and suppresses the phosphorylation of AKT, mTOR, S6 in hiPSC-ECs. **(G)** Immunoblot analysis indicated that ZM 323881 treatment did not affect SND1, AKT, and mTOR in hiPSC-ECs.

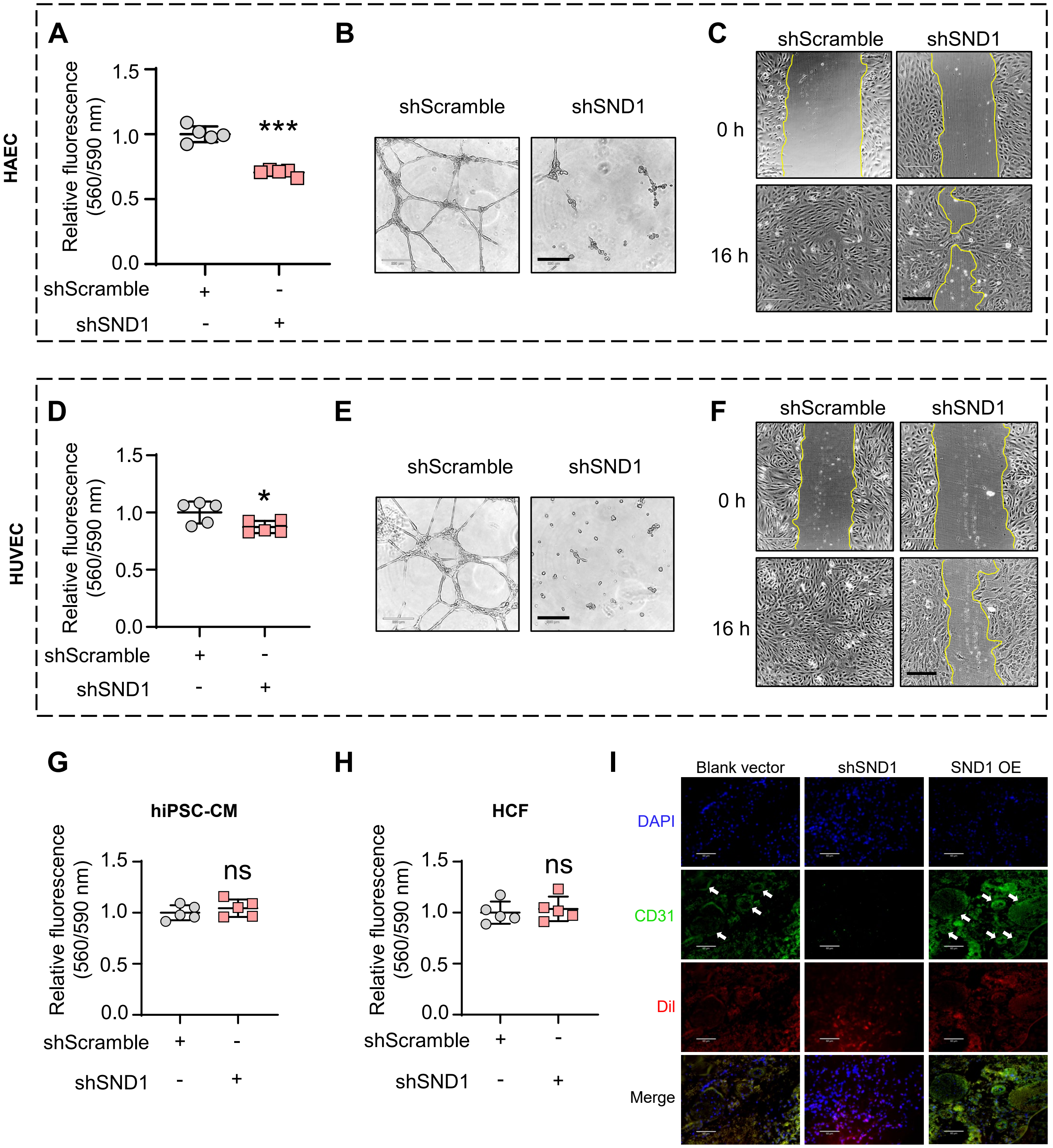

Supplemental Figure 4

Supplemental Figure 4. SND1 knockdown impaired endothelial cell viability and function. (A-C) The effects of SND1 knockdown (KD) on HAEC viability and function was determined by PrestoBlue viability assay (n=5 technical replicates), tube formation assay and scratch assay. Two-tailed Student's t test. Data are presented as mean ± SD. ***P<0.001. Scale bar, 220 µm. **(D-F)** The effects of SND1 KD on HUVEC viability and function was determined by PrestoBlue viability assay (n=5 technical replicates), tube formation assay and scratch assay. Two-tailed Student's t test. Data are presented as mean ± SD. *P<0.05. Scale bar, 220 µm. **(G)** The effects of SND1 KD on hiPSC-CM viability was determined by PrestoBlue viability assay. Two-tailed Student's t test. ns; not significant. Data are presented as mean ± SD. N=5 technical replicates. **(H)** The effects of SND1 KD on HCF viability was determined by PrestoBlue viability assay. N=5 technical replicates. Two-tailed Student's t test. Data are presented as mean ± SD. ns; not significant. **(I)** Representative confocal micrographs of the explanted plugs (related to Fig 4M-O) stained with human anti-CD31 (green) or cell tracking dye (red). Nuclei stained by DAPI. Scale bar=60 µm.

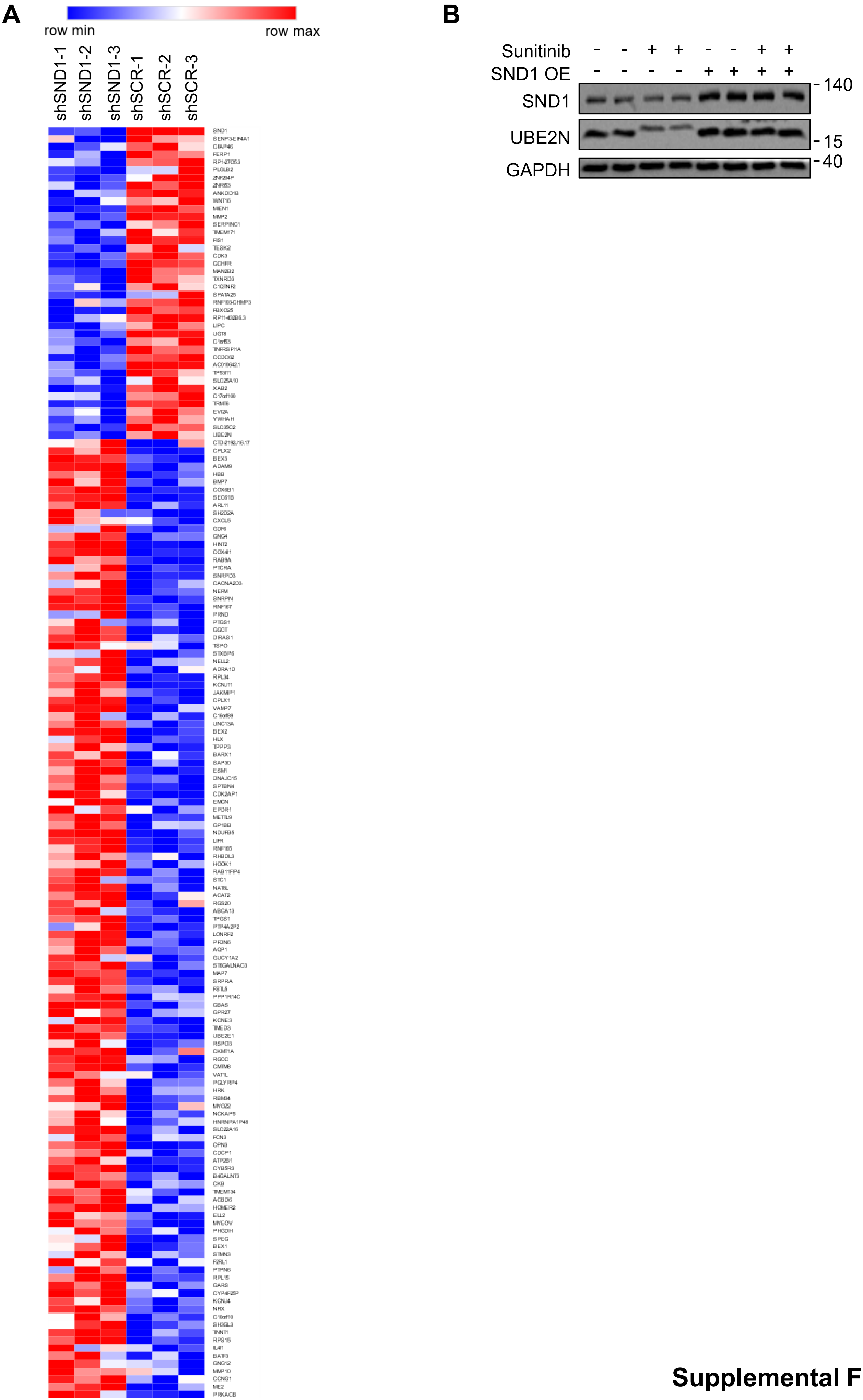

Supplemental Figure 5

Supplemental Figure 5. SND1 regulates UBE2N expression in hiPSC-ECs. (A) Heat map plot of differentially expressed genes (DEGs) in hiPSCs-ECs expressing shSND1s (shSND1-1 to -3) versus shScrambles (shSCR-1 to -3) **(B)** hiPSC-ECs were transduced with virus overexpressing SND1 (SND1 OE) or control. Following exposure to sunitinib (2 μM) or DMSO for 48 hrs, protein expressions of SND1 and UBE2N were monitored by immunoblotting.

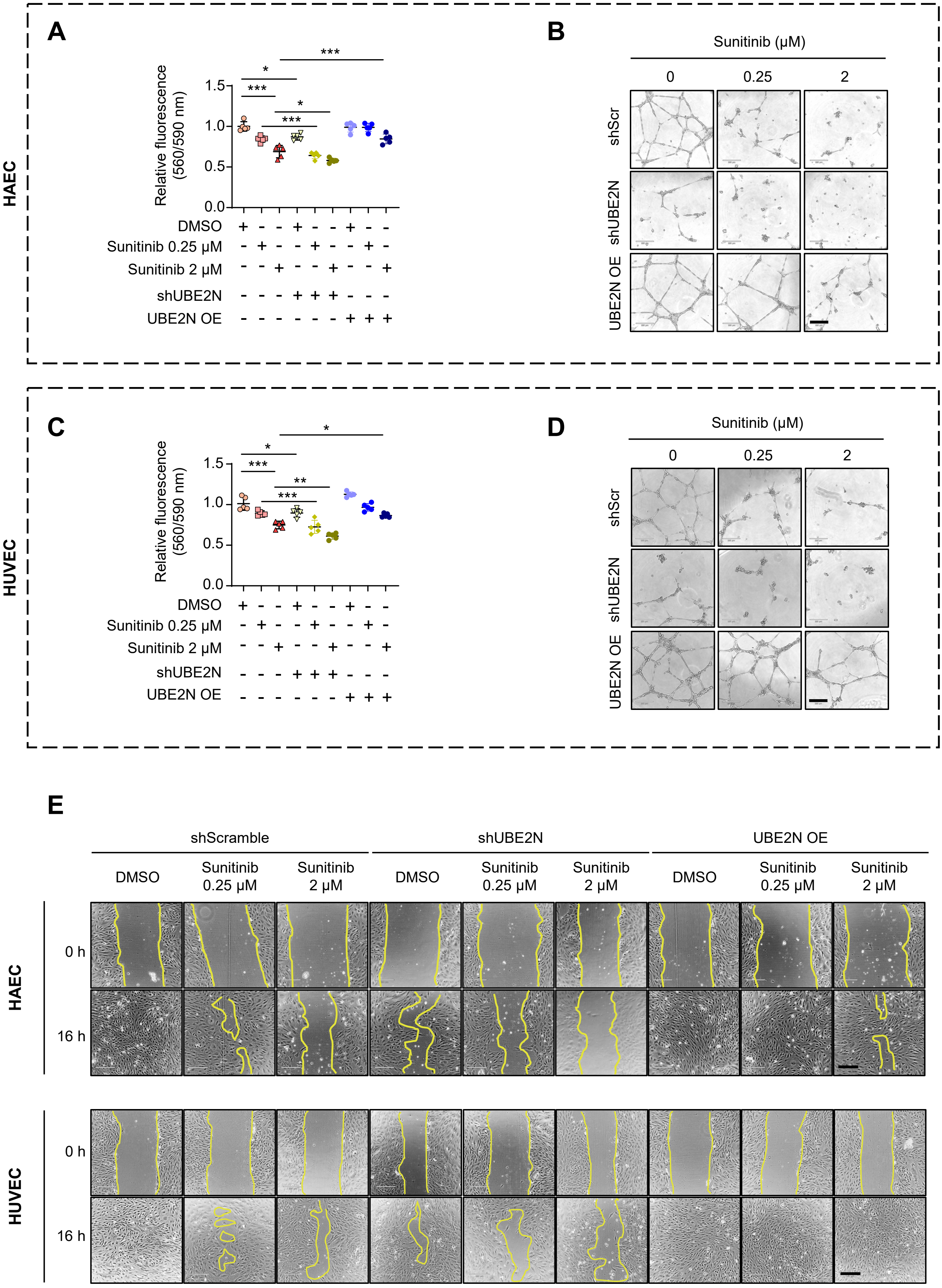

Supplemental Figure 6

Supplemental Figure 6. UBE2N regulates primary endothelial cell function. (A and **B)** The effects of shUBE2N or UBE2N overexpression (OE) on sunitinib-induced HAEC dysfunction was determined by viability assay (n=5 technical replicates) and tube formation assay. One-way ANOVA. Data are presented as mean ± SD. *P<0.05, ***P<0.001. Scale bar, 220 μm. **(C** and **D)** The effects of shUBE2N or UBE2N OE on sunitinib-induced HUVEC dysfunction was determined by viability assay (n=5 technical replicates) and tube formation assay. One-way ANOVA. Data are represented as mean ± SD. *P<0.05, *P<0.01, ***P<0.001. Scale bar, 220 μm. **(E)** Wound healing assay revealed the role of UBE2N on sunitinib-induced endothelial dysfunction. Scale bar, 220 μm.

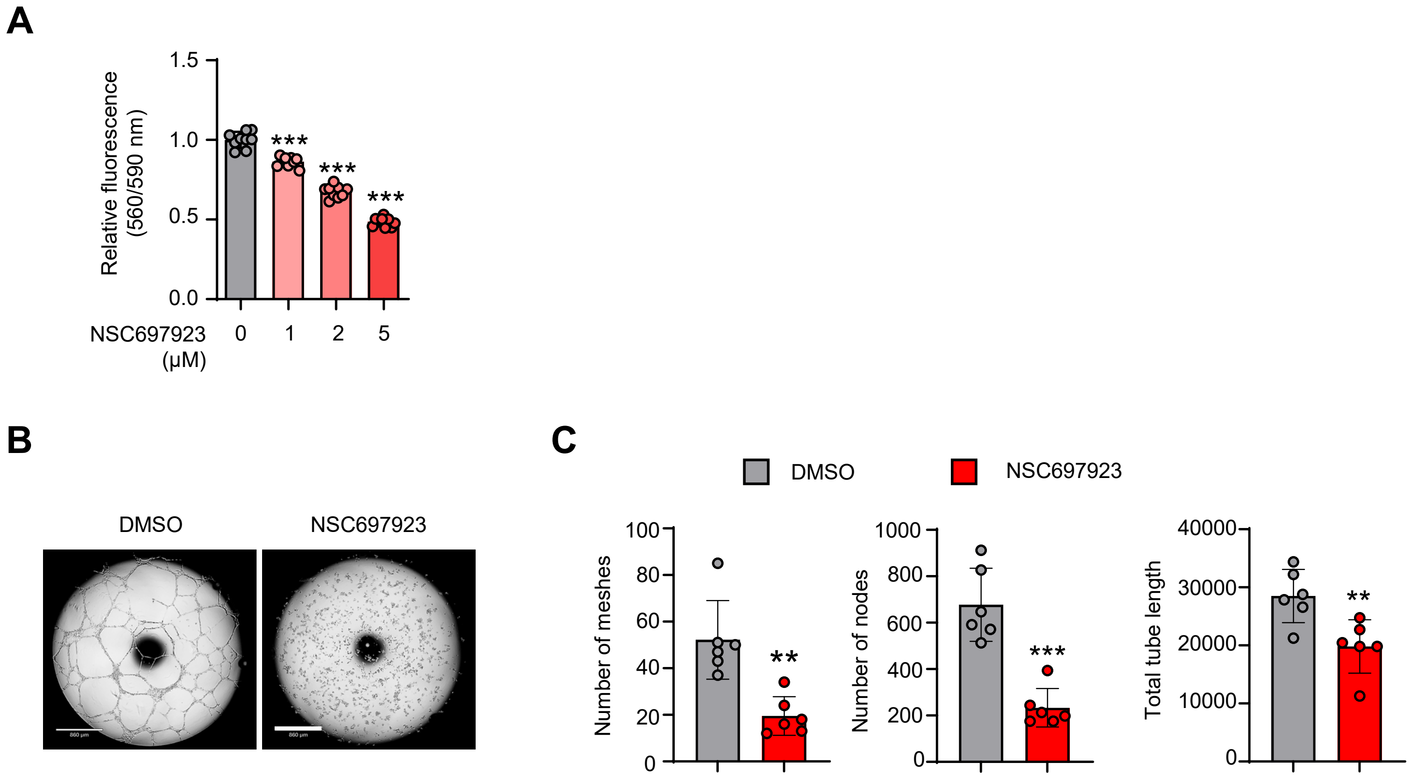

Supplemental Figure 7

Supplemental Figure 7. Inhibition of UBE2N by NSC697923 induced endothelial cell dysfunction. (A) hiPSC-ECs were treated with different concentrations of NSC697923, a selective inhibitor of UBE2N. The effects of NSC697923 on hiPSC-EC viability was determined by PrestoBlue viability assay. One-way ANOVA. Data are represented as mean ± SD. ***$P<0.001$. N=9 replicates from the differentiation of three individual hiPSC lines. **(B** and **C)** The effects of NSC697923 (1 μM) on hiPSC-EC function was determined by tube formation assay. N=6 replicates from the differentiation of two individual hiPSC lines. Scale bar, 860 μm. Two-tailed Student's t test. Data are represented as mean ± SD. **$P<0.01$, ***$P<0.001$.

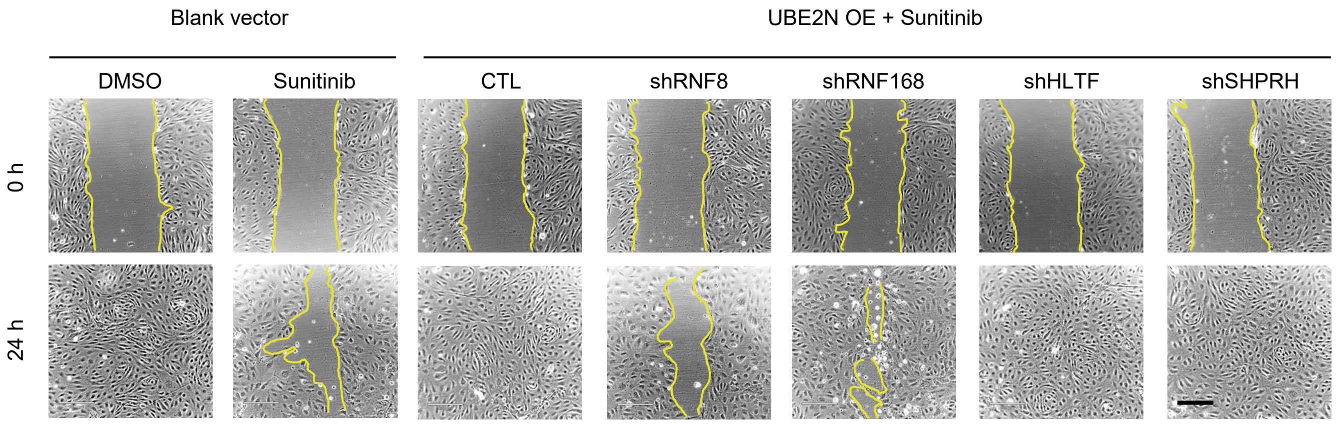

Supplemental Figure 8

Supplemental Figure 8. The protective effects of UBE2N on sunitinib-induced endothelial dysfunction were reversed by knockdown of RNF8 or RNF 168. Wound healing assay showed the protective effects of UBE2N OE on sunitinib-induced endothelial dysfunction were reversed by inhibition of RNF8 or RNF 168 but not HLTF and SHPRH. The yellow line indicates the edges of the scratch wound. Scale bar, 220 μm.

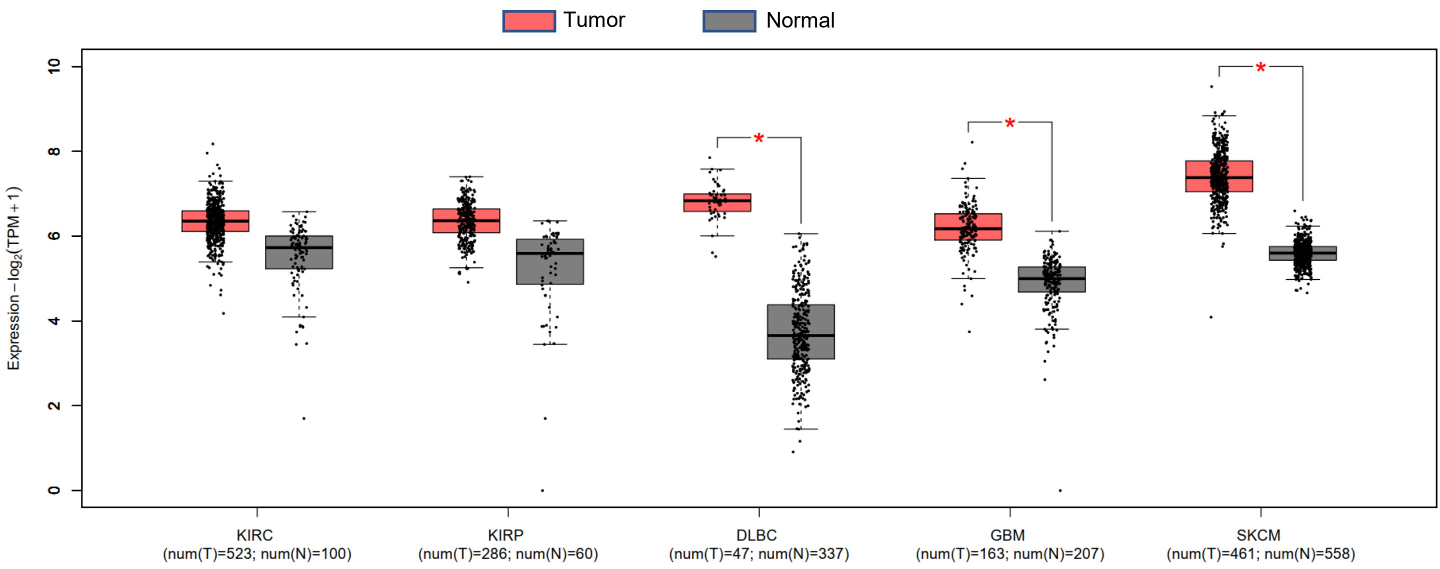

Supplemental Figure 9

Supplemental Figure 9. Analysis of the correlation between SND1 and several cancer types in TCGA. The enrichment of SND1 was found in DLBC, GBM, SKCM, but not KIRC and KIRP, which are cancers that sunitinib is commonly used for treatment. KIRC: Kidney renal clear cell carcinoma; KIRP: Kidney renal papillary cell carcinoma; DLBC: Lymphoid neoplasm diffuse large B-cell lymphoma; GBM: Glioblastoma multiforme; SKCM: Skin cutaneous melanoma.

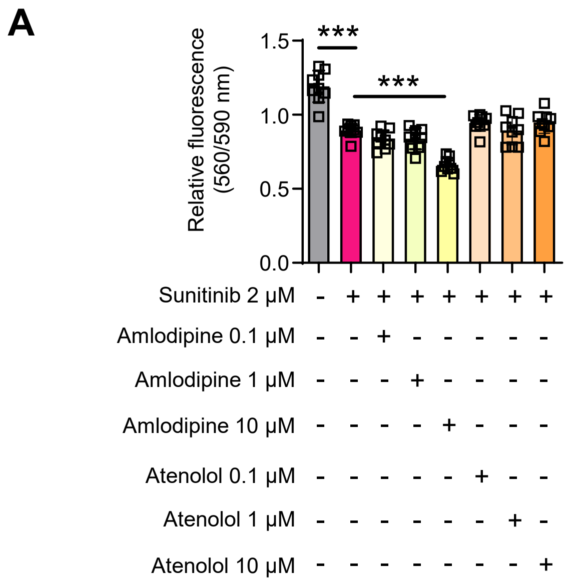

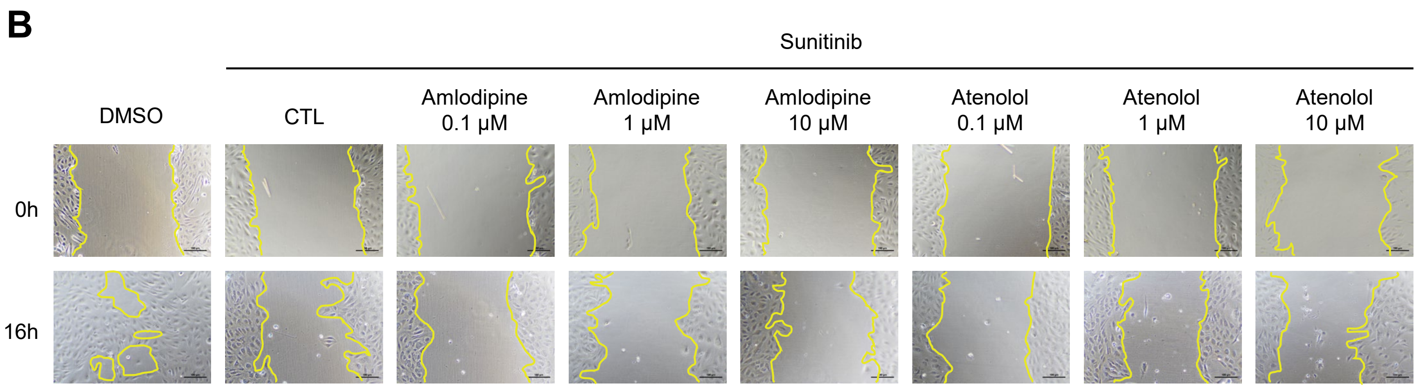

Supplemental Figure 10

Supplemental Figure 10. The effect of antihypertensive drugs Amlodipine and Atenolol on sunitinib-induced endothelial cell dysfunction. (A) hiPSC-ECs were treated with sunitinib (2 μM) for 48 hrs in presence or absence of increasing concentrations of amlodipine or atenolol. Cells treated with DMSO were used as control. Cell viability was determined using the PrestoBlue cell viability reagent. One-way ANOVA. Data are represented as mean ± SD. ***P<0.001. N=9 replicates from the differentiation of three individual hiPSC lines. **(B)** Representative images of wound healing ability of hiPSC-ECs in response to treatment with amlodipine or atenolol along with sunitinib (2 μM) for 48 h. Scale bar, 100 μm.

A

Compound	Ramipril	Lisinopril	Enalapril	Benazepril
Pubchem CID	5362129	5362119	5388962	5362124
Molecular formula	$C_{23}H_{32}N_2O_5$	$C_{21}H_{31}N_3O_5$	$C_{20}H_{28}N_2O_5$	$C_{24}H_{28}N_2O_5$
2D Structure	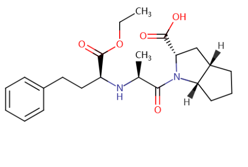	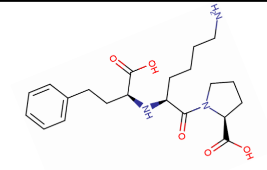	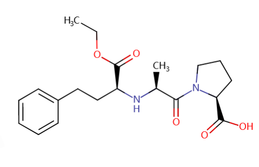	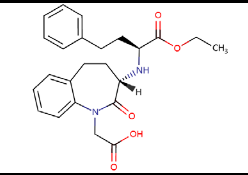
3D Structure	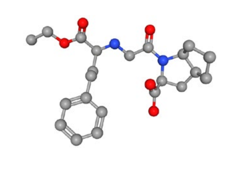	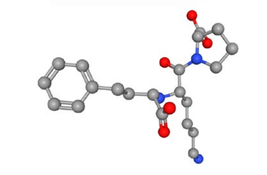	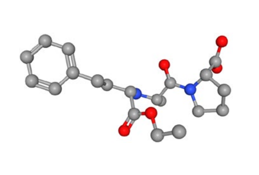	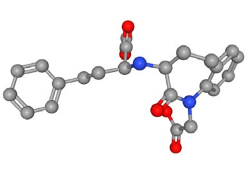

B

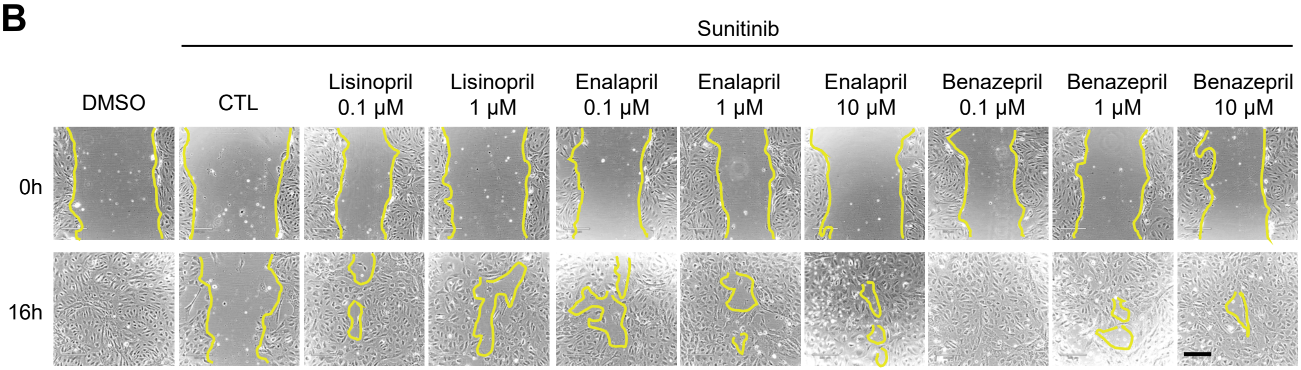

C

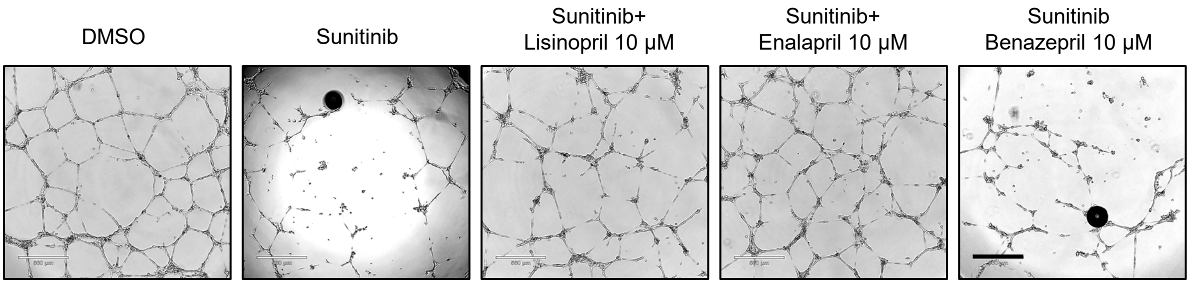

Supplemental Figure 11

Supplemental Figure 11. ACE inhibitors protect against sunitinib-induced endothelial dysfunction. (A) Molecular formula and structure of 4 commonly used ACE inhibitors (ramipril, lisinopril, enalapril, and benazepril). **(B)** The effect of ACE inhibitors on endothelial cell function was determined using wound healing assay. Scale bar, 220 μm. **(C)** The effect of ACE inhibitors on endothelial cell function was determined using tube formation assay. Scale bar, 560 μm.
