# Supplementary material for: Translational regulation of SND1 governs endothelial homeostasis during stress
Source: J Clin Invest. 2025 Feb 3;135(3):e168730. doi: 10.1172/JCI168730 (PMC11785924; doi:10.1172/JCI168730)

Full unedited gel for Figure 2

Figure 2H

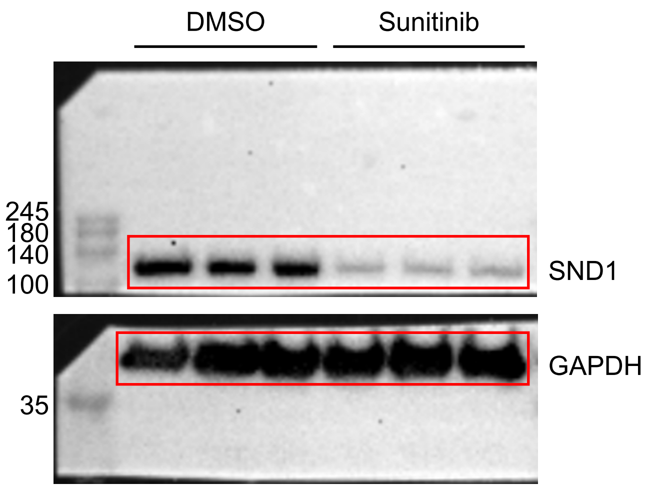

Figure 2N

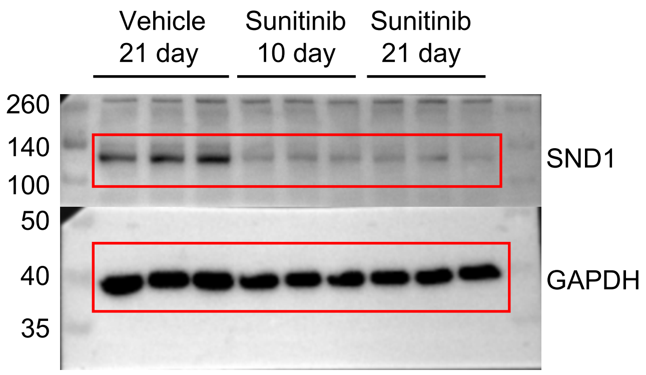

Full unedited gel for Figure 3A, 3C, 3E, 3F

Figure 3A

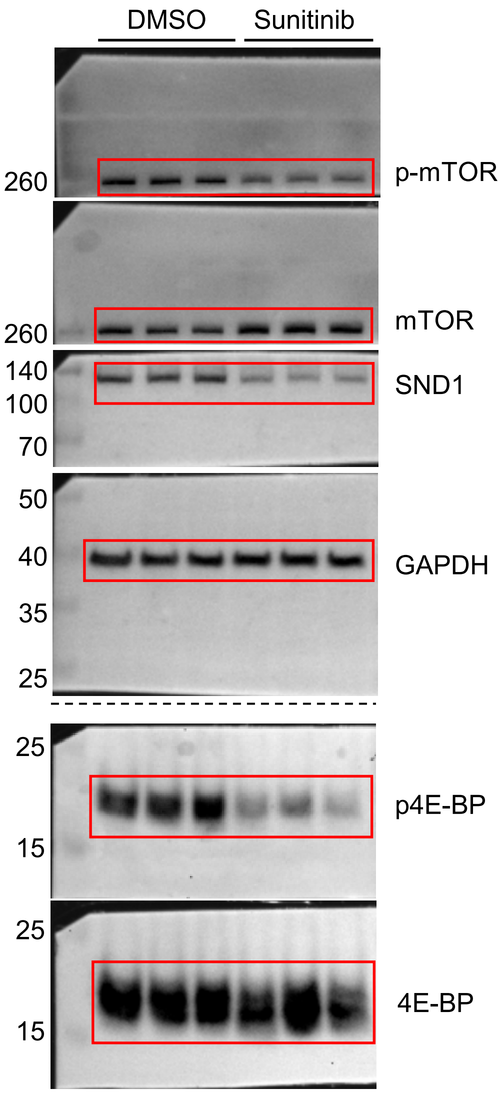

Figure 3C

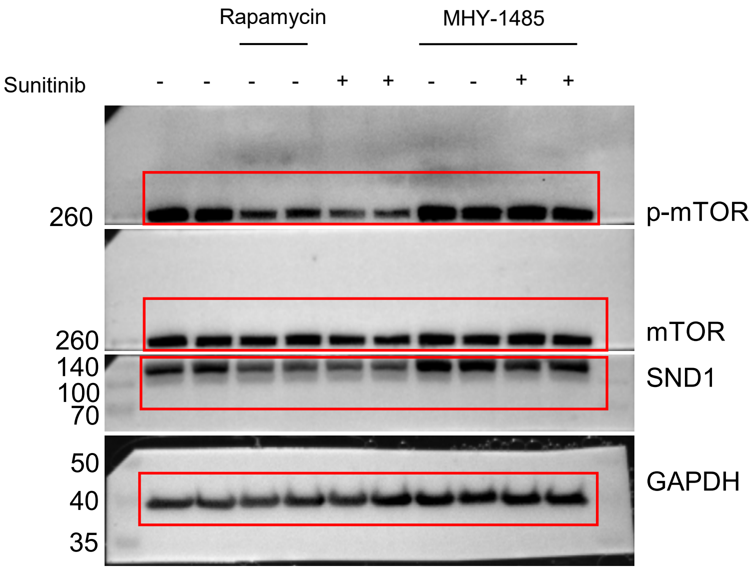

Figure 3E

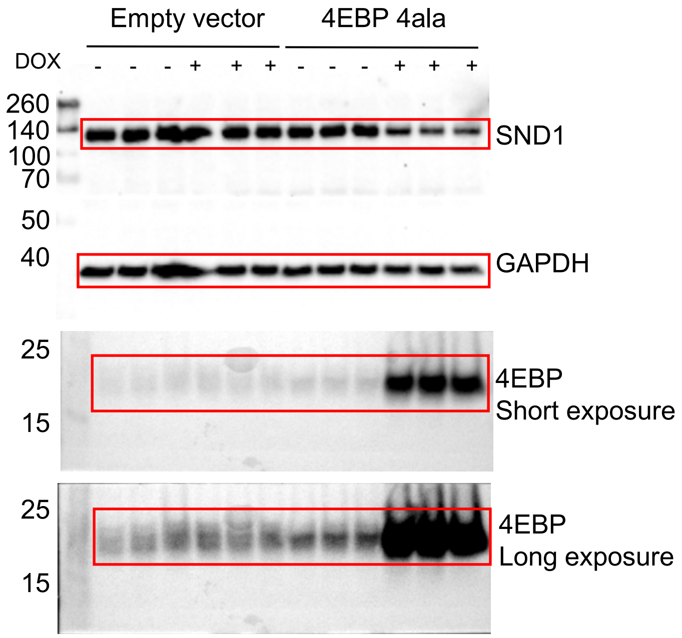

Figure 3F

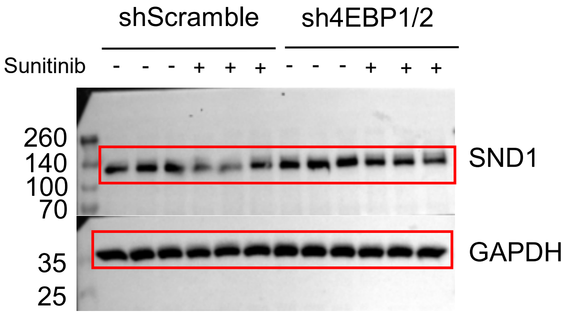

Full unedited gel for Figure 3H, 3I, 3J

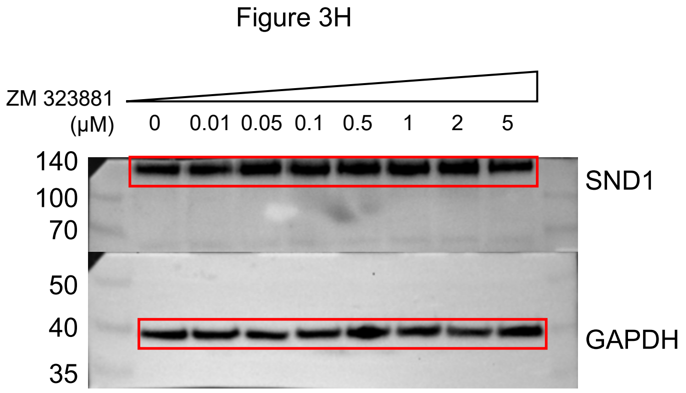

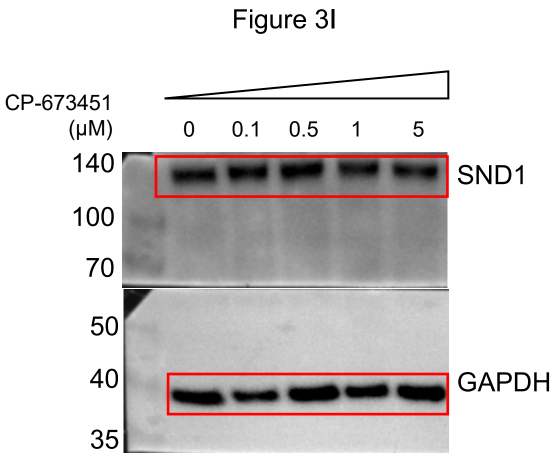

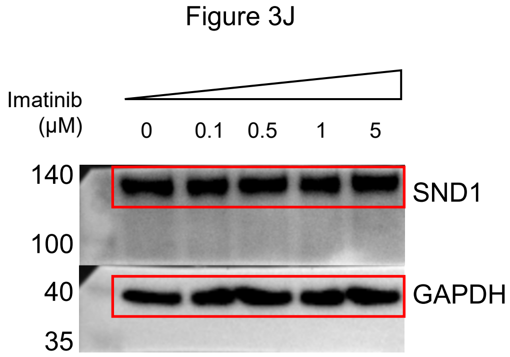

Full unedited gel for Figure 4

Figure 4A

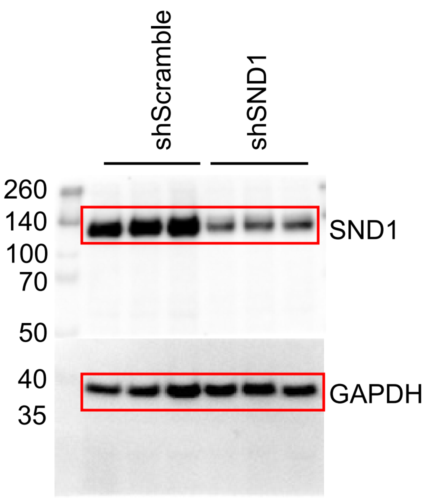

Figure 4B

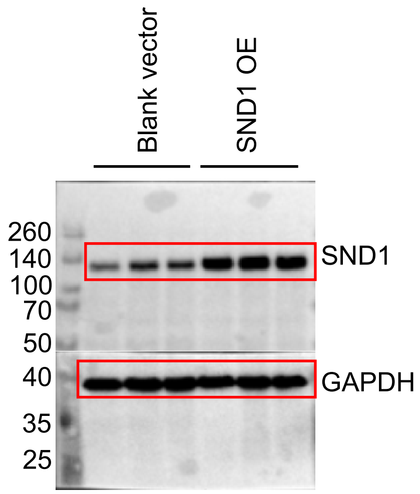

Figure 4O

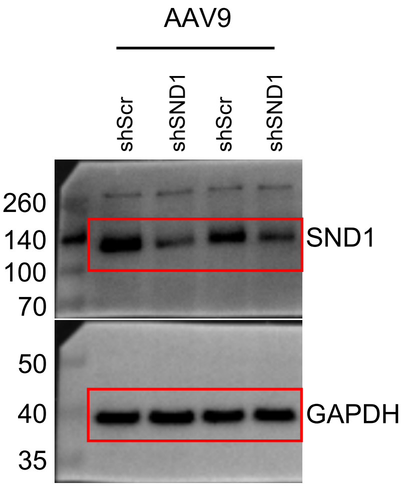

Full unedited gel for Figure 5B, 5D, 5E

Figure 5B

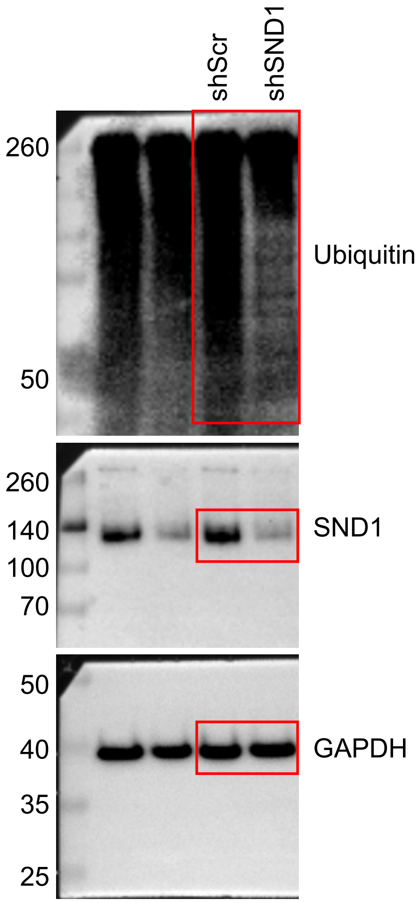

Figure 5D

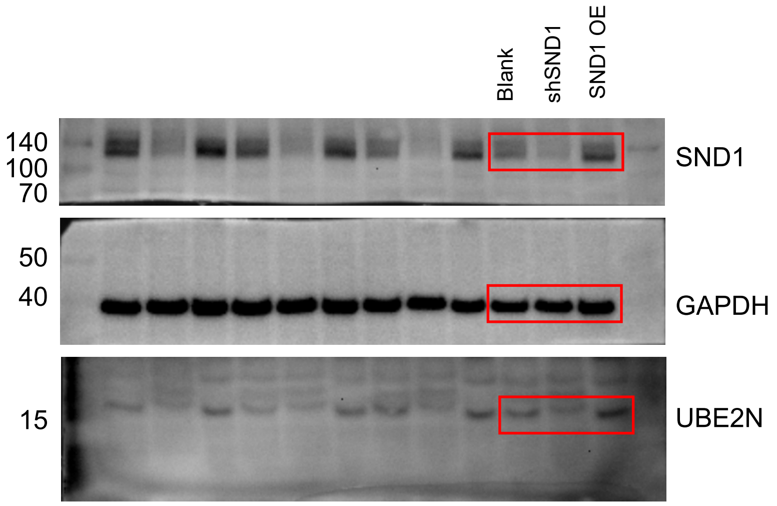

Figure 5E

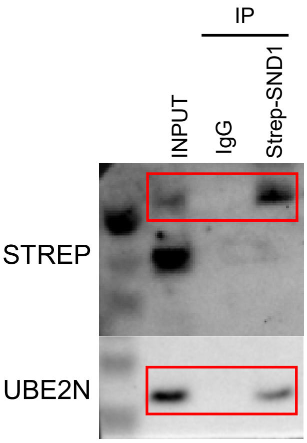

Figure 5E

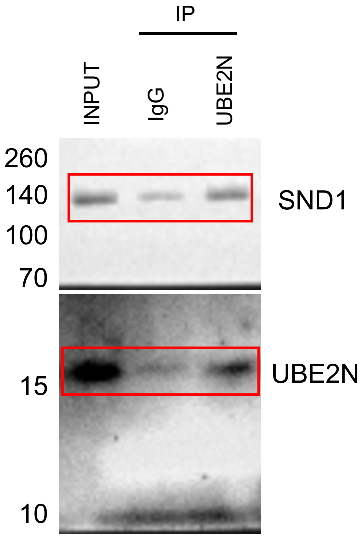

Full unedited gel for Figure 5H, 5K

Figure 5H left

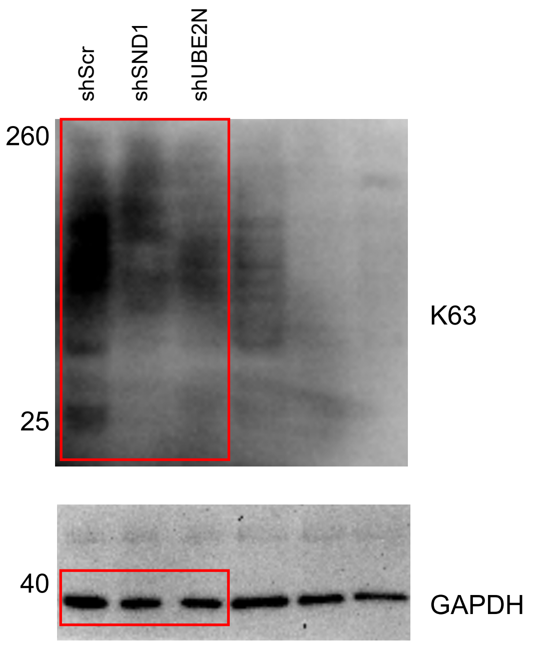

Figure 5H right

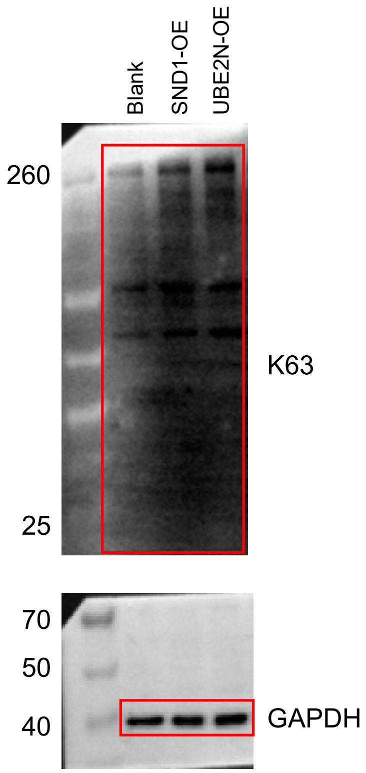

Figure 5K

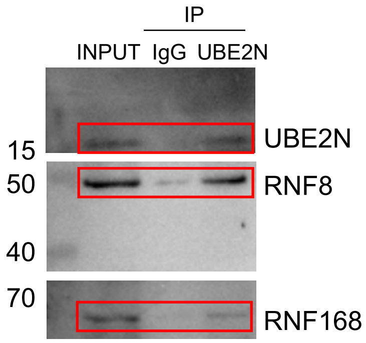

Full unedited gel for Figure 7

Figure 7K

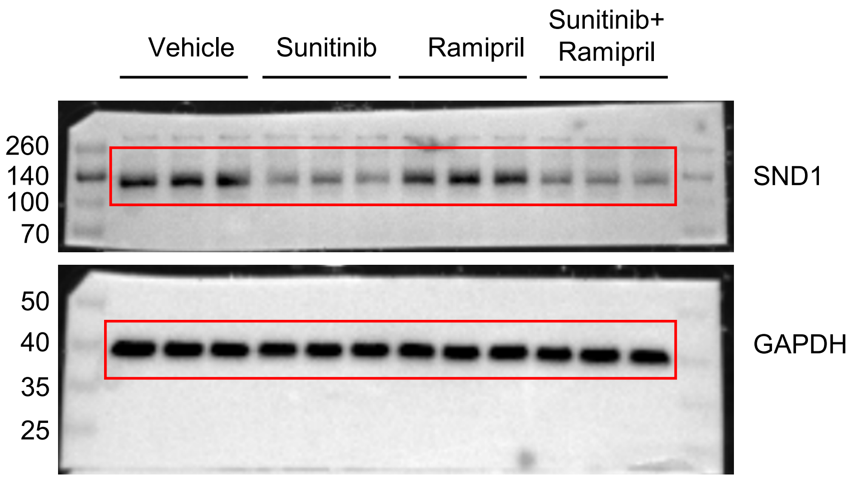

Full unedited gel for Figure S1

Figure S1B

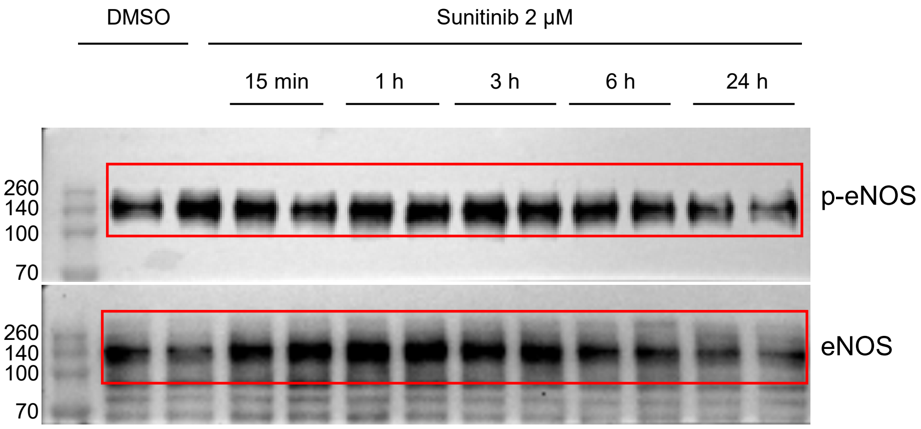

Full unedited gel for Figure S2

Figure S2B

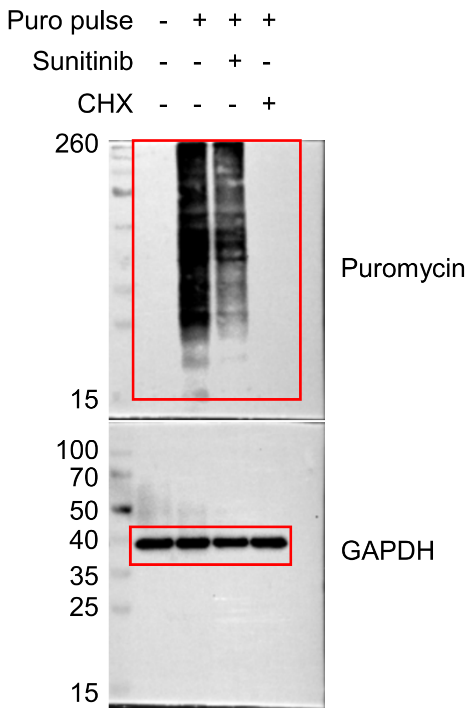

Figure S2E

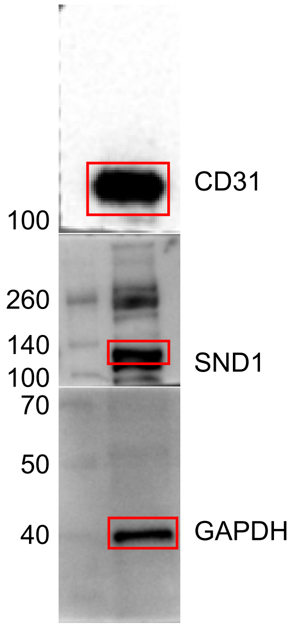

Figure S2G

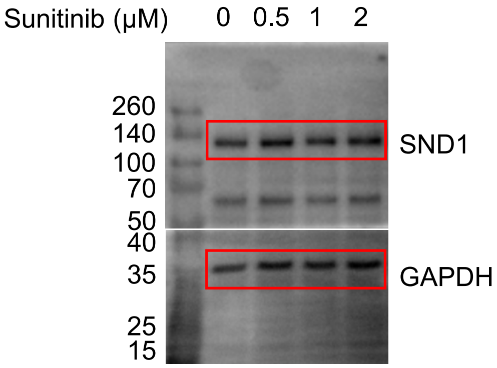

Figure S2H

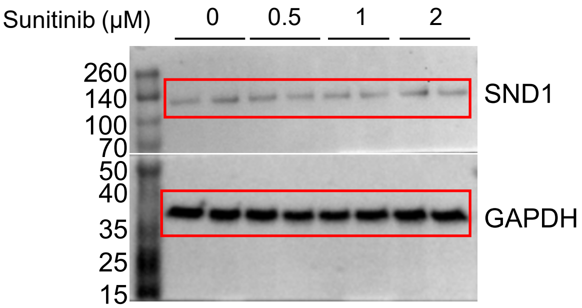

Full unedited gel for Figure S3A, S3B

Figure S3A

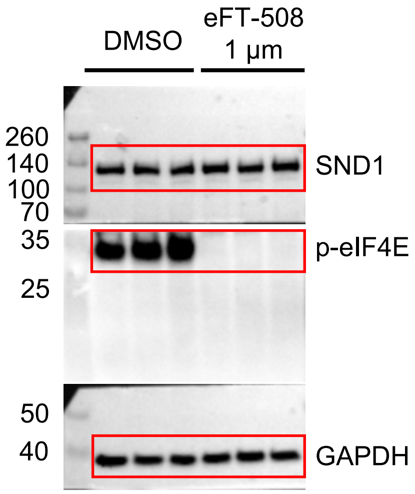

Figure S3B left

hiPSC-EC

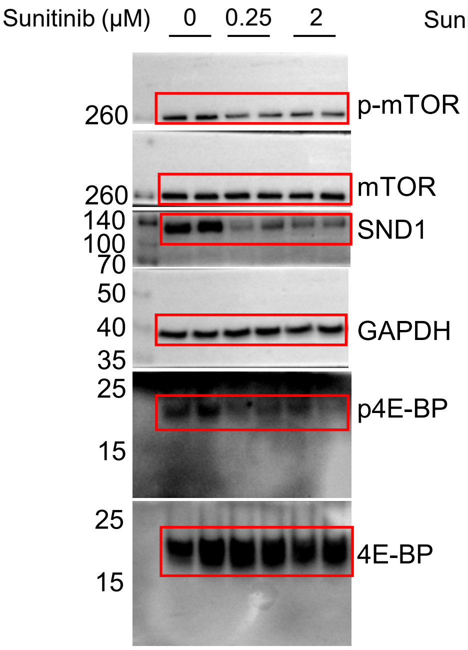

Figure S3B middle

HUVEC

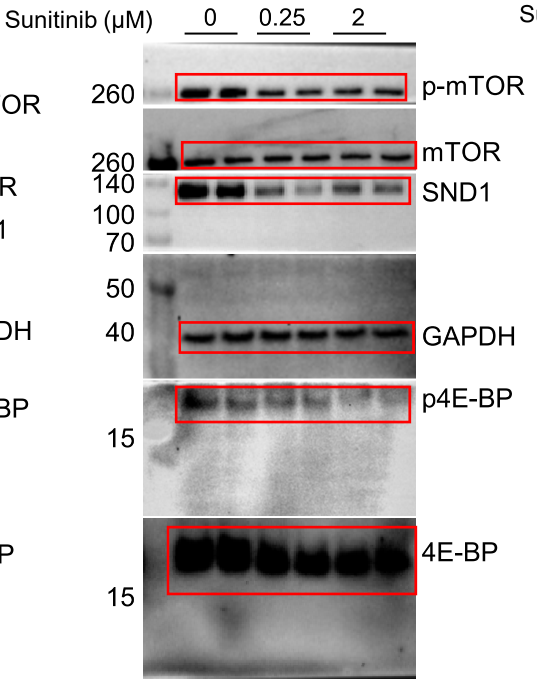

Figure S3B Right

HAEC

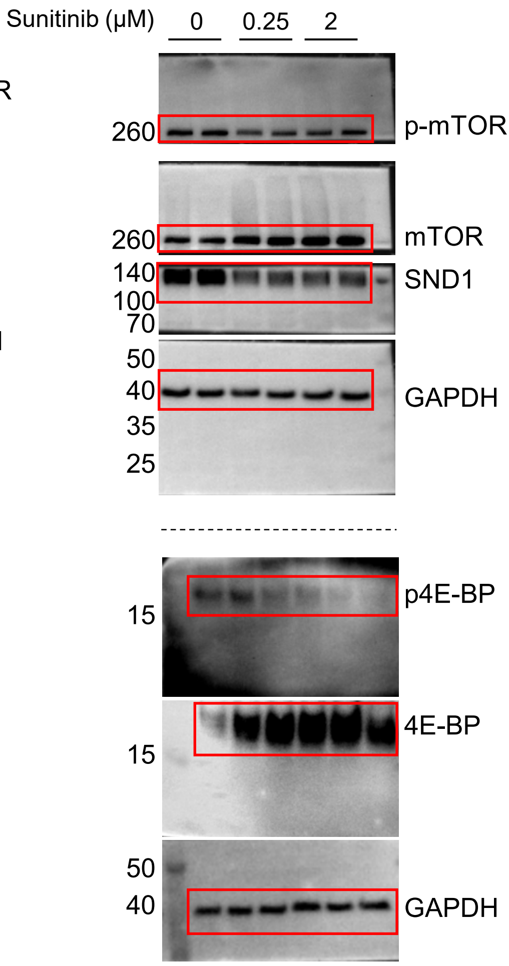

Full unedited gel for Figure S3, C and D

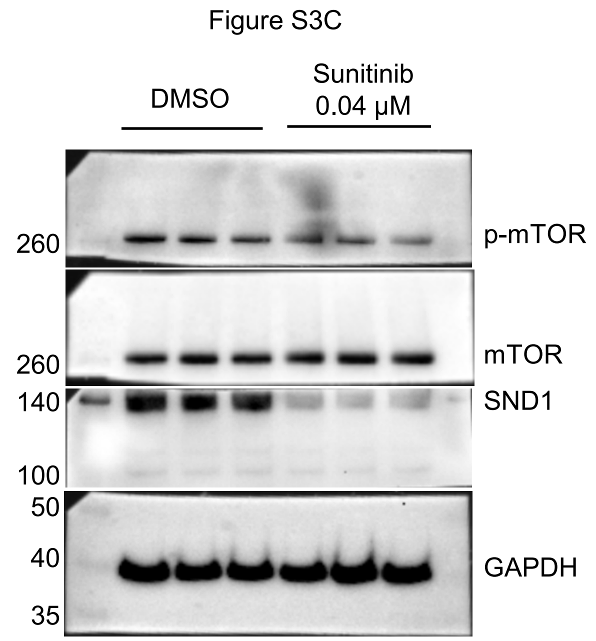

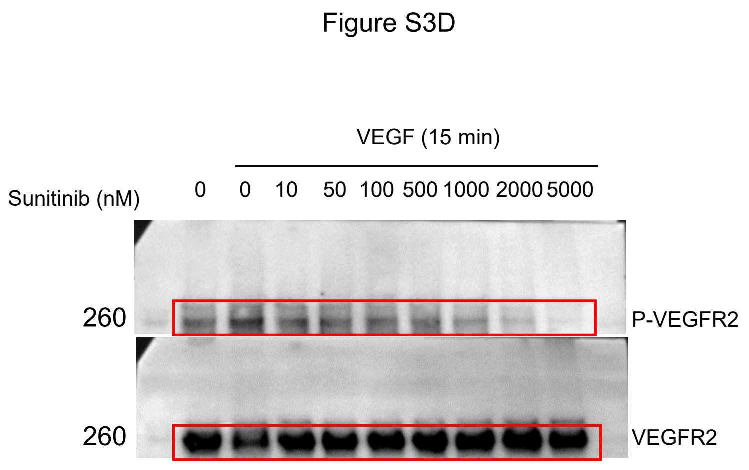

Full unedited gel for Figure S3E

Figure S3E

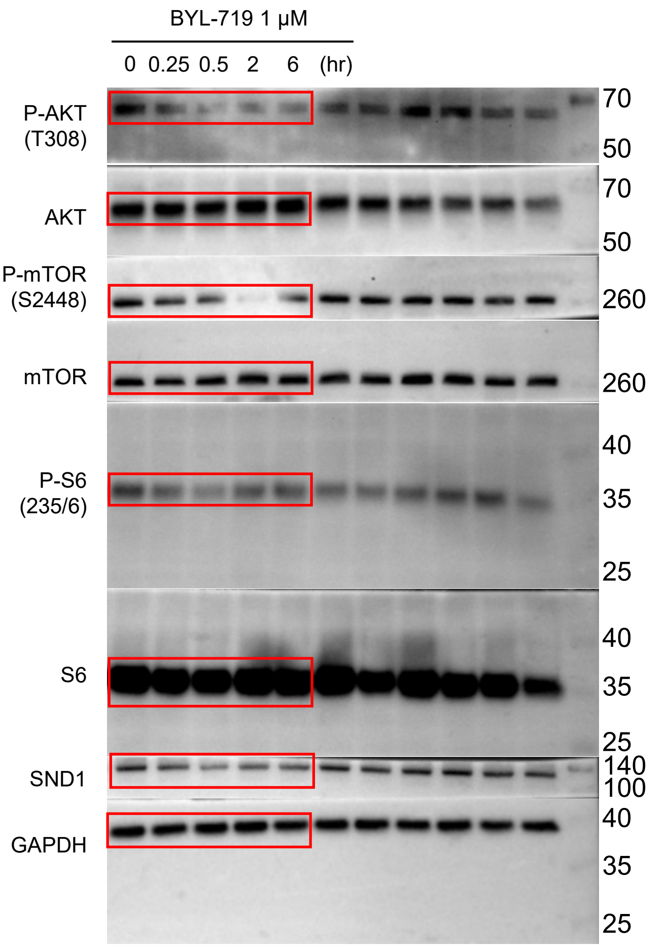

Full unedited gel for Figure S3F

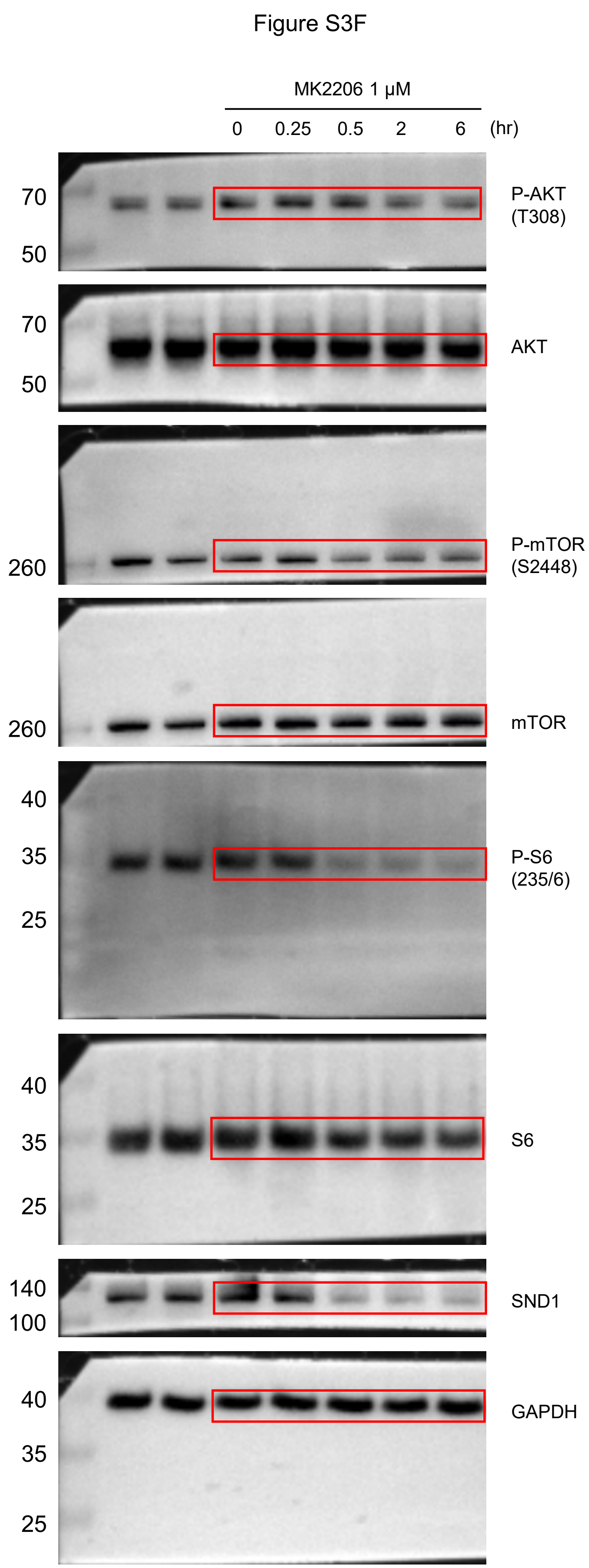

Full unedited gel for Figure S3G

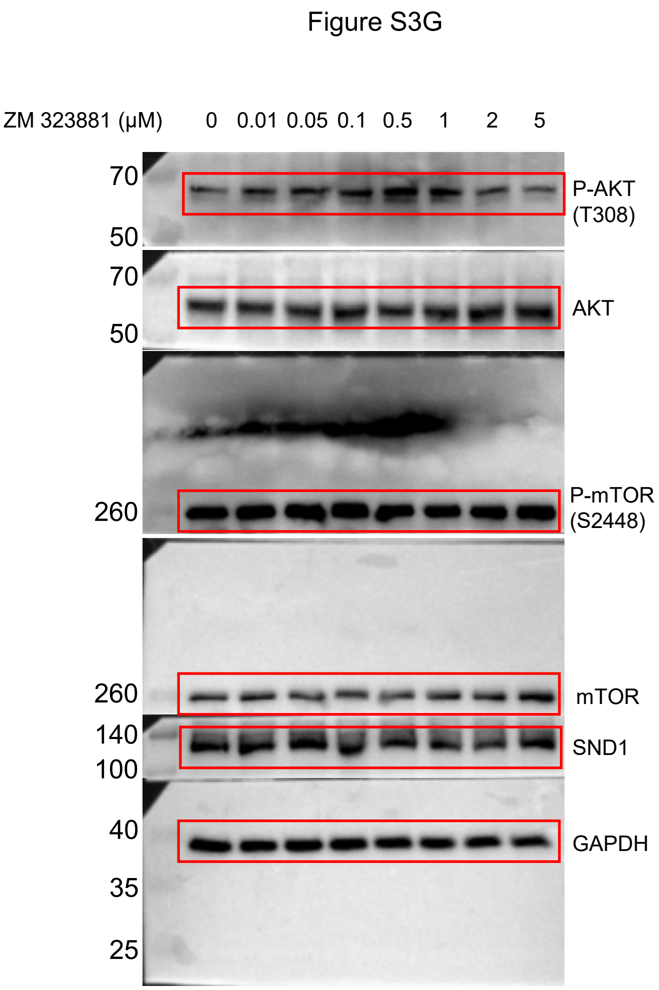

Full unedited gel for Figure S5

Figure S5B

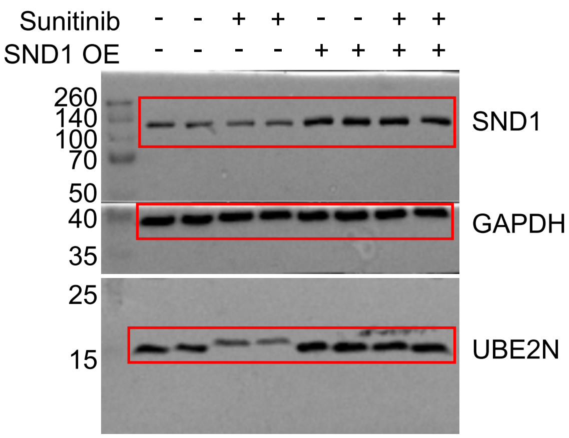

Supplement: Unedited blot and gel images [file jci-135-168730-s028.pdf]
